# Factors Associated with Physical Activity and Sedentary Behavior in Older Adults from Six Low- and Middle-Income Countries

**DOI:** 10.3390/ijerph15050908

**Published:** 2018-05-03

**Authors:** Cadeyrn J. Gaskin, Liliana Orellana

**Affiliations:** Biostatistics Unit, Faculty of Health, Deakin University, Geelong 3220, Australia; cadeyrn.gaskin@deakin.edu.au

**Keywords:** physical activity, sedentary behavior, exercise, older adults, ageing, ecological framework, low- and middle-income countries

## Abstract

Rising life expectancy in low- and middle-income countries (LMIC), coupled with the increasing burden of non-communicable diseases, accentuates the importance of generating information to support public health strategies. With this aim in mind, the purpose of this study was to identify correlates of physical activity and sedentary behavior in LMIC. We analyzed Wave 1 data (collected 2007–2010) from the World Health Organization’s longitudinal Study on global AGEing and adult health (SAGE), which focuses on nationally representative samples of adults aged 50 years and older from six countries (China, *n* = 13,157; India, *n* = 6560; Mexico, *n* = 2301; Russian Federation, *n* = 3763; South Africa, *n* = 3836; and Ghana, *n* = 4305). Associations of physical activity (operationalized as meeting physical activity guidelines of ≥150 min/week of moderate-to-vigorous physical activity or not) and sedentary behavior (≥4 h/day versus <4 h/day) with demographic, health and health risk, functional, interpersonal, and environmental factors were assessed using multivariate logistic models. Across the six countries, we found fairly consistent and reasonably strong associations between both physical activity and sedentary behavior and several demographic factors (age and employment, in particular), self-reported health, instrumental activities of daily living, factors relating to socializing, and household location. Correlates of physical activity and sedentary behavior in LMIC appear to be similar to those found in high-income countries.

## 1. Introduction

Non-communicable disease age-standardized death rates in low- and middle-income countries (LMIC; 756 per 100,000 for men and 565 per 100,000 for women in 2008) exceed those of high-income countries (65% and 85% higher for men and women, respectively) [1]. The negative effects of globalization, rapid unplanned urbanization, and increases in sedentary living mean that the burden of non-communicable diseases in these countries will probably grow at an increasingly faster pace [1]. These trends underpin the importance of global targets for the prevention and control of non-communicable diseases, such as a 10% relative reduction in the prevalence of insufficient physical activity [2]. Analysis of self-reported data from 51 mainly LMIC has shown that one-fifth of adults aged 50–59 (men: 17.1%; women: 21.3%) and a quarter of those aged 60–69 (men: 22.3%; women: 28.6%) could be classified as physically inactive [3]. Knowledge of the correlates of physical activity and sedentary behavior in older adults living in LMIC could assist with the implementation of initiatives to increase physical activity and reduce sedentary behavior in these countries.

Demographic and biological correlates of physical activity in LMIC (most studies have been conducted in Brazil and China) are consistent with those found in high-income countries [4]. In LMIC, males and younger people tend to be more active than females and older adults, respectively [4,5]. Evidence is equivocal on the relationship between wealth and physical activity, with some studies (mainly from Brazil and China) showing the wealthier are more active [4], whereas a recent study from Bangladesh showed that people regarding themselves as poor were more active than those classifying themselves as rich [5]. Those with greater social support are also likely to be physically active [4,6]. Environmental correlates of physical activity include urbanicity (those living in rural areas tend to be more active) [5,7], ownership of various household devices (inverse association with television, car, and computer ownership) [8,9], and the built environment (e.g., density of bus stations and access to bike paths) [10]. Studies focusing on older adults in a broader number of LMIC are still needed.

Much less is known about the correlates of sedentary behavior in LMIC. In one Chinese study involving adults aged 30–79 with no history of major disease, sedentary leisure time was positively associated with body mass index (BMI) values, waist circumference, and body fat, after controlling for age, study area, education, and annual household income [11]. These findings are largely consistent with those found in (mainly) high-income countries [12]. Evidence is limited, however. More research is warranted to explore the correlates of sedentary behavior in LMIC.

With increasing life expectancy in LMIC [13], attention needs to be paid to the health and well-being of growing populations of older adults. A greater understanding of the correlates of physical activity and sedentary behavior in LMIC may assist in designing interventions for specific subpopulations in these countries. To this end, we investigated the correlates of physical activity and sedentary behavior in adults aged 50 and older from six LMIC (China, India, Mexico, Russian Federation, South Africa, and Ghana). Specifically, we assessed the extent to which physical activity and sedentary behavior were associated with demographic, health and health-risk, functional, interpersonal, and environmental factors.

## 2. Materials and Methods

### 2.1. Design

Wave 1 data from the World Health Organization’s (WHO) longitudinal Study on global AGEing and adult health (SAGE) were used for this secondary cross-sectional analysis. SAGE uses multistage cluster sampling strategies in the collection of data from nationally-representative samples of adults aged 50 years and older from six countries (China, India, Mexico, Russian Federation, South Africa, and Ghana), as well as smaller, comparative samples of adults aged 18–49 [14]. The six countries provide a diverse representation of geographic regions, levels of economic development, and stages in demographic and health transitions, as well as including the two countries with the highest populations in the world. These six countries were classified as LMIC when data collection commenced in 2007 [15]. Details on the study methods have been published [14,16]. Briefly, each household was allocated to one of two mutually exclusive categories: (1) 50+ households and (2) 18–49 households. Face-to-face interviews were conducted from 2007 to 2010. For each household, one household questionnaire was completed, and all persons aged 50 and over were invited to participate in individual interviews. In all countries, a standardized instrument, methods, interviewer training, and translation protocols were used. The World Health Survey team led the translation of the instrument, based on WHO guidelines (refer to http://www.who.int/substance_abuse/research_tools/translation/en/ [17]). This method involves forward translation, expert panel back-translation, pretesting and cognitive interviewing, and the development of a final version. SAGE received approval from the WHO Ethical Review Committee and the respective committees in each participating country. Written informed consent was obtained from all study participants. The SAGE dataset is publicly available upon request (http://www.who.int/healthinfo/sage/en/ [18]).

For our analysis, data from the 50+ households were used. The overall sample sizes were 13,157 for China, 6560 for India, 2301 for Mexico, 3763 for Russian Federation, 3836 for South Africa, and 4305 for Ghana. Response rates were high in China (92% for adults aged 50–59 and 93% for those aged 60 and older), India (90%, 85%), Russian Federation (80%, 84%), South Africa (76%, 80%), and Ghana (76%, 80%), and lower in Mexico (42%, 55%) [14]. Potential correlates of physical activity or sedentary behavior, based on research literature or reason, were selected from the SAGE dataset. Consistent with previous work on the correlates of physical activity and sedentary behavior [4,12,19], we used an ecological framework to group the factors. Such frameworks position the individual within an ecosystem, depicting interactions between factors proximal and distal to the individual. Here, we grouped factors as follows: demographic, health and health risk, functional, interpersonal, and environmental.

### 2.2. Measures

#### 2.2.1. Demographic Factors

Self-reported factors were age (categorized as 50–59, 60–69, 70–79, and 80+ years), sex (male and female), education completed (never been to school, less than primary school, primary school, secondary/high school, and college or more), and employment (working, not working, and retired/too old to work). Household wealth (country-specific quintiles) was derived from household ownership of selected durable goods, dwelling characteristics, and access to services [20].

#### 2.2.2. Health and Health-Risk Factors

BMI was computed from weight and height measurements and categorized as: underweight (<18.5 kg/m^2^), normal weight (18.5 to <25 kg/m^2^), overweight (25 to <30 kg/m^2^), and obese (≥30 kg/m^2^) [21,22,23]. Self-reported factors were alcohol use (never drunk; drunk in the past; one drink per day or less, on average; and more than one drink per day, on average), smoking and tobacco use (no, less than daily, and daily), number of non-communicable diseases (summed from self-reports of arthritis, stroke, angina, diabetes, chronic lung disease, asthma, hypertension, and cataracts), pain (Over the last 30 days how much of bodily aches or pains did you have? Response options: *none*, *mild*, *moderate*, *severe*, and *extreme*), and self-rated health (In general, how would you rate your health today? Response options: *very good*, *good*, *moderate*, *bad*, and *very bad*).

#### 2.2.3. Functional Factors

Mobility/dismobility was assessed using a 4 m timed walk. Participants were instructed to walk at their normal walking pace over a flat, straight, obstacle-free surface. Dismobility was defined as a speed of ≤0.6 m/s [24]. Visual acuity was measured using myopic and hyperopic tests with LogMAR charts (Tumbling “E” Chart for 4 m testing and Tumbling “E” Near Vision Card for 40 cm testing. Precision Vision Ltd., 944 First Street, LaSalle, IL 61301, USA). Corrected vision for each eye was tested separately, with the result from the eye with the better vision used for the analysis. The results for distance vision (4 m) and near vision (40 cm) were categorized (*mild or no impairment* and *moderate impairment or greater*) using accepted criteria [25]. Verbal learning and memory were assessed using the WHO/UCLA Auditory Verbal Learning Test [26], for which various scoring options are possible [27]. Over three trials, participants were read the same list of 10 words and asked to recall as many words as they remembered. The mean number of recalled words over the three trials was used for the analysis (words recalled immediately). Participants were then involved in unrelated cognitive and physiological tests for approximately 10 min, before being asked to recall the 10 words again (this time without the list of words being read to them). The difference between the mean number of recalled words over the first three trials and the number of recalled words following the delay was used for the analysis (words lost with delay). That is, if the mean number of words recalled for the first three trials was 9 and the number of words recalled following the delay was 7, then the number of words lost with the delay is 2 (i.e., 9 − 7 = 2). Difficulties with activities of daily living (ADL, 23 activities; e.g., sitting for long periods) and instrumental activities of daily living (IADL, 5 activities; e.g., taking care of household responsibilities) over the last 30 days were assessed using a 5-point Likert scale anchored with *none* and *extreme/cannot do*. The mean ADL and IADL scores were used for the analysis. Disability was measured using the screener version (12 items) of the World Health Organization Disability Assessment Schedule 2.0 (WHODAS 2.0) [28,29]. Simple scoring was used, because it is a more valid method than applying weights to the WHODAS 2.0 items [30]. Scores were transformed to a 100-point scale, where higher values indicate greater disability. Quality of life was assessed using the short, 8-item version of the WHO quality of life scales [31], with scores transformed to a 100-point scale, where higher values signify greater quality of life.

#### 2.2.4. Interpersonal Factors

Emotional loneliness was measured as a single item (Did you feel lonely for much of the day yesterday? Response options: yes and no). Four questions focused on social participation—How often in the last 12 months have you: (1) had friends over to your home; (2) been in the home of someone who lives in a different neighborhood than you do or had them in your home; (3) socialized with coworkers outside of work; and (4) gotten out of the house/your dwelling to attend social meetings, activities, programs or events or to visit friends and relatives? The response options were *never*, *once or twice per year*, *once or twice per month*, and *once or twice per week or more*. Marital status was categorized as coupled (married or cohabitating) or uncoupled (separated, divorced, widowed, or never married).

#### 2.2.5. Environmental Factors

Location was classified as urban (an area legally proclaimed as being urban, including towns, cities, and metropolitan areas) or rural (an area not classified as urban). Access to personalized motorized transport (defined as the possession of a car, motorbike, or both modes of transport by anyone in the household) and the presence of a computer in the household (response options: *yes* and *no*) were self-reported. Safety on the street after dark (How safe do you feel when walking down your street alone after dark?) and safety when at home (In general, how safe from crime and violence do you feel when you are alone at home?) were both self-reported using a 5-point Likert scale anchored with *completely safe* and *not at all safe*.

#### 2.2.6. Physical Activity and Sedentary Behavior

The Global Physical Activity Questionnaire (GPAQ, version 2) was used to measure participation in physical activity and sedentary behavior [32]. The instrument assesses frequency (days in a typical week) and duration (hours and minutes in a typical day) of moderate and vigorous physical activity in each of three domains (work, travel, and recreation), as well as time spent in sedentary behaviors (sitting or reclining) on a typical day. Studies of concurrent validity have shown highly variable correlations between GPAQ scores (e.g., moderate-to-vigorous physical activity and sedentary behavior) and both accelerometer and pedometer data, but stronger associations with scores on the International Physical Activity Questionnaire [33,34]. Test–retest reliability has generally found to be adequate [33,34,35]. A binary variable indicating whether or not participants had met WHO physical activity guidelines (150 min/week or more of moderate-to-vigorous physical activity for adults aged 18–64 years and those aged 65 years and older [36]) was defined. Sedentary behavior was dichotomized as ≥4 h/day and <4 h/day. Sitting for ≥4 h/day is associated with increased risk of all-cause mortality, except for those engaged in about 50–65 min or more of moderate intensity physical activity (or equivalent) per day [37].

### 2.3. Analysis

We conducted the analysis using SAS (version 9.4). For each country, we ran separate analyses for physical activity and sedentary behavior. Some of the factors were omitted from the analyses due to large amounts of missing or implausible data: education (South Africa), BMI (Mexico, Russian Federation, and South Africa), mobility (Russian Federation and South Africa), distance and near vision (Mexico and Russian Federation), number of words lost with delay (South Africa), and socializing with coworkers outside of work (South Africa). Multicollinearity was assessed among the variables in each set of factors (demographic, health and health risk, functional, interpersonal, and environmental factors) in a linear regression with time spent sedentary (in minutes) as the outcome. In each country, the scores for IADL, ADL, and disability were highly correlated (variance inflation factor >4). IADL (but neither ADL nor disability) was included in the adjusted models because IADL was more strongly related to both physical activity and sedentary behavior than the other two variables (ADL and disability). No further factors showed strong collinearity. Due to the large number of factors to be investigated, and following an ecological framework, we adopted a three-stage modeling approach as in Solomon et al. [38]. First, univariate logistic regressions were fitted with each independent factor as the sole covariate. Independent factors associated with the outcome variables (*p* < 0.10) were selected for the next phase of the analysis. The demographic factors were included in all multivariate models. Specifically, age, sex, and employment were always included, along with either or both of education and household wealth (both were retained if both *p* < 0.10, otherwise the variable with the smaller *p* value was retained). Second, we ran *partially adjusted* logistic regressions with each set of factors (e.g., health and health-risk factors) as predictors. Only those variables retained from the first stage of the analyses and the demographics factors were used in this second phase. Third, we performed *fully adjusted* logistic regressions with each of the factors retained from the second phase. The results for the unadjusted and fully adjusted regressions are reported. Analyses were run on the subsets of adults with complete data on the factors included in the fully adjusted models.

## 3. Results

### 3.1. Physical Activity

In the physical activity models, the sample sizes of adults with complete data on factors included in the models were 11,046 for China (84% of SAGE participants aged 50 years and older), 5816 for India (89%), 2045 for Mexico (89%), 3441 for Russian Federation (91%), 2975 for South Africa (86%), and 3752 for Ghana (87%). The percentages of adults meeting physical activity guidelines in each sample varied among the six countries: China (32%), India (41%), Mexico (27%), Russian Federation (48%), South Africa (21%), and Ghana (59%).

The descriptive statistics for adults meeting physical activity guidelines are provided in Table 1. The odds ratios estimated under unadjusted models including demographic, health and health risk, functional, interpersonal, and environmental factors are shown in Table S1. The estimates for the fully adjusted models are provided in Table 2. The description of results focuses predominantly on the fully adjusted models.

#### 3.1.1. Demographic Factors

Across the six countries, age, employment, and household wealth were the variables most consistently associated with meeting physical activity guidelines. The odds of meeting guidelines significantly decreased with age in all countries. Not working and being retired/too old to work were both significantly associated with lower odds of meeting guidelines in five countries (China, India, Mexico, South Africa, and Ghana), with the results for Russian Federation in the same direction. Adults from households in the lowest wealth quartiles were more likely to be meeting guidelines in China, India, South Africa, and Ghana. In Russian Federation, the reverse was found, however, whereby adults from lower income households had lower odds of meeting guidelines.

The relationship between sex and meeting physical activity guidelines was equivocal. Females were more likely to be meeting guidelines in India and Russian Federation, males were more likely to be meeting guidelines in Ghana, and no differences between the sexes were found for China, Mexico, and South Africa.

Education was related to meeting physical activity guidelines in China, India, and Ghana, but the directions of the associations were not consistent across countries.

#### 3.1.2. Health and Health-Risk Factors

Poorer ratings of health were associated with being less likely to be meeting physical activity guidelines in India, Russian Federation, South Africa, and Ghana. In three countries (China, South Africa, and Ghana), people reporting pain had higher odds of meeting guidelines (these results contrast with the unadjusted estimates, where higher pain severity was associated with lower odds of meeting guidelines). Among the countries in which BMI was considered in the analysis (China, India, and Ghana), being overweight or obese (compared with normal weight) was related to lower odds of meeting physical activity guidelines in China and Ghana. Few significant relationships were found for meeting guidelines and alcohol use (China and Russian Federation), smoking and tobacco use (India and South Africa), and number of non-communicable diseases (China).

#### 3.1.3. Functional Factors

Increased difficulties with IADL were associated with lower odds of meeting guidelines in all countries except Russian Federation (for which IADL was not selected for the final model). Other functional factors associated with meeting physical activity guidelines were mobility (China and India), distance vision (China and India), and verbal learning and memory (words recalled immediately; India, South Africa, and Ghana).

#### 3.1.4. Interpersonal Factors

Factors measuring social connectedness were generally associated with increased odds of meeting physical activity guidelines. Adults who more frequently had friends visiting their homes (Mexico and Ghana), were visiting people in different neighborhoods or having them coming to visit (Russian Federation and Ghana), were socializing with coworkers outside of work (China, India, and Ghana), and were engaging in social activities outside of home (China, India, and Russian Federation) were more likely to be meeting guidelines. In contrast, adults visiting people in different neighborhoods or having them come to visit in Mexico were less likely to be meeting guidelines (this result was in the opposite direction to the corresponding univariate estimates, however). Adults who were coupled were more likely to be meeting guidelines (Mexico and Ghana). No significant relationships were found between meeting guidelines and emotional loneliness.

#### 3.1.5. Environmental Factors

Meeting physical activity guidelines was more frequent among older adults living in rural areas in China, India, Russian Federation, and Ghana. Those who perceived themselves to be completely or very safe when home alone had higher odds of meeting guidelines than those who perceived themselves to be moderately safe or slightly safe/not at all safe (India, South Africa, and Ghana). Perceived safety out on the street after dark was associated with meeting guidelines in China and Ghana but the direction of the relationship was inconsistent. Adults reporting the presence of computers in their households were less likely to be meeting guidelines (China, Russian Federation, and Ghana). Meeting guidelines was associated with access to motorized transport only in India and Ghana.

### 3.2. Sedentary Behavior

For the sedentary behavior models, the sample sizes of adults with complete data on factors included in the models were 11,355 for China (86% of SAGE participants aged 50 years and older), 6171 for India (94%), 2052 for Mexico (89%), 3446 for Russian Federation (92%), 3033 for South Africa (88%), and 3900 for Ghana (91%). The percentages of adults sedentary for ≥4 h/day varied between the six samples: China (45%), India (38%), Mexico (21%), Russian Federation (58%), South Africa (37%), and Ghana (43%).

The descriptive statistics for adults sedentary for ≥4 h/day are provided in Table 3. The odds ratios estimated under unadjusted models including demographic, health and health risk, functional, interpersonal, and environmental factors are shown in Table S2. The estimates for the fully adjusted models are provided in Table 4. The description of results focuses predominantly on the fully adjusted models. 

#### 3.2.1. Demographic Factors

Across countries, age and employment were the demographic factors most consistently associated with sedentary behavior. Increased age was related to higher odds of ≥4 h/day sedentary behavior in all countries except South Africa (although the association was in the same direction). Not working and being retired/too old to work were associated with higher odds of sedentary behavior compared with working (China, India, South Africa, and Ghana).

In some countries relationships were observed between ≥4 h/day sedentary behavior and sex (India and Mexico, where women were less likely to be sedentary), education (China, Mexico, and Russian Federation), and household wealth (China, India, and South Africa).

#### 3.2.2. Health and Health-Risk Factors

Poorer self-rated health was associated with higher odds of ≥4 h/day sedentary behavior in China, Russian Federation, and Ghana (conversely, and in contrast to their respective univariate results, in India and South Africa, adults with poorer self-rated health had lower odds of ≥4 h/day sedentary behavior). Among the countries in which BMI was considered in the analysis (China, India, and Ghana), higher BMI was related to greater odds of ≥4 h/day sedentary behavior in China and Ghana. Few relationships were found between ≥4 h/day sedentary behavior and alcohol use (India, Russian Federation, and Ghana), smoking and tobacco use (India), and number of non-communicable diseases (China and India). No associations were present between sedentary behavior and pain.

#### 3.2.3. Functional Factors

No functional factors were significantly related to ≥4 h/day sedentary behavior in more than three countries. Adults with dismobility had higher odds of ≥4 h/day sedentary behavior in China, India, and Mexico. Those with increased difficulties with IADL had higher odds of ≥4 h/day sedentary behavior in China, Mexico, and South Africa. Higher quality of life was associated with lower odds of ≥4 h/day sedentary behavior in India, South Africa, and Ghana.

Inconsistent relationships were present between ≥4 h/day sedentary behavior and verbal learning and memory factors (China, Mexico, and South Africa). Vision impairment was associated with higher odds of ≥4 h/day sedentary behavior in China (near vision) and South Africa and Ghana (distance vision).

#### 3.2.4. Interpersonal Factors

People reporting emotional loneliness had higher odds of ≥4 h/day sedentary behavior in Mexico, Russian Federation, and Ghana. More frequent visits from friends was associated with increased odds of ≥4 h/day sedentary behavior for China and Ghana (a different association pattern was observed for South Africa, however, with more frequent visits related to lower odd of sedentary behavior). Increased frequency of visiting people in different neighborhoods or them coming to visit was related to lower odds of ≥4 h/day sedentary behavior for China, India, and Ghana. More frequent socializing with coworkers outside of work was associated with increased odds of ≥4 h/day sedentary behavior for India and Mexico, but lower odds for Russian Federation and Ghana. Increased frequency of social activities outside of home was related to higher odds of spending ≥4 h/day sedentary for China, South Africa, and Ghana. Being uncoupled was associated with slightly higher odds of ≥4 h/day sedentary behavior in China.

#### 3.2.5. Environmental Factors

Adults in India and South Africa who perceived themselves to be completely or very safe out on the street after dark had higher odds of ≥4 h/day sedentary behavior than those who perceived themselves to be moderately safe or slightly safe/not at all safe. The reverse was observed for Ghana. Those with access to personal motorized transport had lower odds of ≥4 h/day sedentary behavior in China, India, and Russian Federation, with a nonsignificant association in the same direction for South Africa. People in China and Mexico living in rural areas had lower odds of ≥4 h/day sedentary behavior. No significant associations were present between ≥4 h/day sedentary behavior and either computers in household or safety when home alone.

## 4. Discussion

Across the six LMIC, we found fairly consistent and reasonably strong associations (fully adjusted models) between both physical activity and sedentary behavior and demographic factors (age and employment, in particular), self-reported health, IADL, factors relating to socializing, and location of household. Age was the only variable that was related to both physical activity and sedentary behavior in all six countries. Like in previous research [4,12], age had an inverse relationship with physical activity and a positive association with sedentary behavior. Some researchers have speculated that this decline in activity with age may have a biological basis (e.g., reduced dopamine release or loss of dopamine receptors with age, affecting areas of the brain relating to motivation for locomotion) in conjunction with psychosocial factors and the physical environment [39,40,41]. Across countries, older adults who were working were clearly more active than those who were not working or were retired/too old to work. This finding suggests that the work-related physical activity (e.g., active transport to and from work, and occupation-related physical activity) is not replaced with other activity (e.g., leisure time physical activity) for older adults who have retired or are otherwise not working. The associations between work and sedentary behavior are consistent with the findings of studies from (mainly) high-income countries [12].

Of the health and health-risk factors, self-rated health was the factor most consistently linked with physical activity and sedentary behavior. Poorer rated health was associated with lower odds of meeting physical activity guidelines and higher odds of sedentary behavior. The prominence of this factor lends itself to at least two possible interpretations. First, self-reported health may have captured aspects of health not identified through the other factors (e.g., undiagnosed non-communicable diseases and health conditions not included in the SAGE dataset). Second, individuals’ perceptions of health may matter more than the views of researchers (who decide what health conditions are included in surveys) and health practitioners as to what constitutes good health. Using self-rated health measures in surveys enables participants to prioritize and assess different aspects of their health, which is perhaps a more sensitive way of measuring general health [42].

The findings pertaining to functional factors serve to re-emphasize the positive relationship between physical function and physical activity. Across all countries, with the exception of Russian Federation, having fewer difficulties with IADL was associated with increased odds of meeting physical activity guidelines, and in three countries (China, Mexico, and South Africa), greater difficulties with IADL was correlated with sedentary behavior. The relationship between activity and function may be bidirectional (i.e., increased physical activity may improve physical function, and higher levels of physical function may increase a person’s willingness to be physically active). Evidence from studies with community-dwelling older adults is that increased levels of physical activity are associated with fewer difficulties with basic activities of daily living [43]. Equally, greater physical function promotes increased participation in physical activity [44].

Taken collectively, the results for interpersonal factors generally show that more frequent social activity is associated with increased odds of meeting physical activity guidelines and decreased likelihood of spending ≥4 h/day sedentary. In general, these findings would seem to broadly reflect the literature, in which an association between social support and physical activity in older adults has been reported [45]. This relationship would appear to be stronger in some circumstances (e.g., when the social support is for physical activity and comes from family members) than others (e.g., when general social support is provided). In our study, the reasons for the relationships were not evident. Plausible explanations include: (a) participating in social activities required active transport to and from the activities; (b) the social activities involved physical activity; and (c) those who were more physically active may have been more willing and able to attend the social activities.

Of the environmental factors, where an older adult lived (urban versus rural) had the most consistent relationship with physical activity and sedentary behavior. Adults in rural areas were generally more likely to be meeting physical activity guidelines and less likely to be spending ≥4 h/day sedentary. These results confirm previous findings linking urbanization with lower physical activity and greater sedentary behavior [5,7].

Much higher levels of physical inactivity (not meeting physical activity guidelines) were found in the SAGE data than in a previous study of 51 mainly LMIC included in the World Health Survey (2001) [3]. In this World Health Survey study and our study, findings can be compared for people aged 60–69 years. For this age group, the levels of inactivity (the inverse of the findings for meeting physical activity guidelines in Table 1) were higher in our study with SAGE data (China, 68%; India, 62%; Mexico, 68%; Russian Federation, 50%; South Africa, 84%; Ghana, 38%) than in the World Health Survey (22% for men and 29% for women). The researchers using World Health Survey study data recognized the prevalence of physical inactivity among people in this survey was substantially lower than in comparable studies, and suggested instrumentation and the seasons in which data were collected may have influenced results. The results from SAGE data are consistent with evidence from a systematic review that between 20% and 60% of older adults (aged 60 and over) have typically been found to meet physical activity guidelines (i.e., between 40% and 80% are physically inactive) [46].

A small number of associations seemed counterintuitive. Notably, in the fully adjusted models of three countries, people with more severe pain had higher odds of meeting physical activity guidelines. The direction of this relationship was opposite to that found in the unadjusted models. Overadjustment or misspecification of the models may explain these unexpected findings.

This study has several limitations. First, although physical activity questionnaires enable the timely and cost-effective gathering of data in large epidemiological studies, the limitations of such measures are well known [47,48]. Pertinent to our study is the appropriateness of the GPAQ for research with older adults from several LMIC. There is evidence that older adults sometimes find questions on physical activity and sedentary behavior difficult to understand, often resulting in misreporting (overreporting more commonly than underreporting) of activity levels [49]. Such issues may also affect younger adults. The implications of these biases are that some older adults in our study may have been miscategorized (e.g., classified as meeting physical activity guidelines when they, in actuality, performed insufficient moderate and vigorous physical activity to meet the threshold). Such misclassification bias may have attenuated some of the estimated associations. In addition, although the GPAQ has been evaluated and used in many developed and developing countries [33,50], the potential remains for the differences observed between countries to be an artifact of differences between various translations of the GPAQ and how questions were asked, interpreted, and answered. Second, the SAGE survey involved a long interview that might have affected the accuracy of responses, especially in older adults who are frail. Third, this study had a cross-sectional design, meaning that temporality between the variables cannot be established. Fourth, the response rates for Mexico were much lower than for the other five countries, potentially underrepresenting some subpopulations for this country. The lower response rates for Mexico have been attributed to the short time available for fieldwork (data were collected from 2009 to 2010 and multiple visits to respondents’ homes were not made if they were not at home during the initial visit) and a high level of attrition from Wave 0 [14]. Wave 0 in SAGE refers to data collected from the six countries during the 2002/2004 World Health Survey. Even with these limitations, to our knowledge this is the first study to report on the correlates of physical activity and sedentary behavior for representative samples of older adults in several LMIC.

## 5. Conclusions

The central finding from our work is that physical activity and sedentary behavior were fairly consistently related to several demographic factors (age and employment, in particular), self-reported health, IADL, factors relating to socializing, and location of household. Our findings may be useful in targeting interventions towards those who are most likely to be physically inactive. Thought needs to be given to developing interventions that target adults with specific characteristics (e.g., those who are older) and to do so using methods that are sensitive to the resource constraints of LMIC. Further research is necessary to understand how these factors may interact and which of them may be modifiable determinants. We expect that analysis of data from the next two waves of SAGE (when they become available) will yield valuable information in this respect. In the design of future projects, strong consideration should be given to using objective measures of physical activity and sedentary behavior. The use of such measures would strengthen research findings.

## Figures and Tables

**Table 1:** Descriptive statistics for the samples of adults and for those meeting physical activity guidelines (active).

Factors	China		India		Mexico		Russian Federation		South Africa		Ghana	
	Sample	Active	Sample	Active	Sample	Active	Sample	Active	Sample	Active	Sample	Active
	n (%) ^a^	n (%) ^b^	n (%) ^a^	n (%) ^b^	n (%) ^a^	n (%) ^b^	n (%) ^a^	n (%) ^b^	n (%) ^a^	n (%) ^b^	n (%) ^a^	n (%) ^b^
**Demographic Factors**												
Age												
50–59	4941 (45%)	1951 (40%)	2716 (47%)	1401 (52%)	393 (19%)	143 (36%)	1303 (38%)	804 (62%)	1441 (44%)	425 (30%)	1508 (40%)	1037 (69%)
60–69	3320 (30%)	1061 (32%)	1983 (34%)	747 (38%)	845 (41%)	270 (32%)	940 (27%)	473 (50%)	1053 (32%)	173 (16%)	1054 (28%)	650 (62%)
70–79	2248 (20%)	477 (21%)	887 (15%)	204 (23%)	564 (28%)	116 (21%)	871 (25%)	310 (36%)	593 (18%)	68 (12%)	836 (22%)	406 (49%)
80+	537 (5%)	57 (11%)	230 (4%)	26 (11%)	243 (12%)	21 (9%)	327 (10%)	61 (19%)	212 (6%)	19 (9%)	354 (9%)	136 (38%)
Sex												
Male	5109 (46%)	1820 (36%)	2965 (51%)	1267 (43%)	799 (39%)	260 (33%)	1231 (36%)	623 (51%)	1406 (43%)	347 (25%)	1968 (53%)	1277 (65%)
Female	5937 (54%)	1726 (29%)	2851 (49%)	1111 (39%)	1246 (61%)	290 (23%)	2210 (64%)	1025 (46%)	1893 (57%)	338 (18%)	1784 (48%)	952 (53%)
Education completed ^c^												
Never been to school	2672 (24%)	849 (32%)	2922 (50%)	1138 (39%)	408 (20%)	100 (25%)			^d^			
Less than primary school	2047 (19%)	793 (39%)	647 (11%)	267 (41%)	850 (42%)	231 (27%)					2432 (65%)	1488 (61%)
Primary school	2256 (20%)	845 (38%)	846 (15%)	381 (45%)	436 (21%)	126 (29%)	369 (11%)	103 (28%)			404 (11%)	241 (60%)
Secondary/high school	3607 (33%)	1006 (28%)	1401 (24%)	592 (42%)	185 (9%)	60 (32%)	2397 (70%)	1192 (50%)			916 (24%)	500 (55%)
College or more	464 (4%)	53 (11%)			166 (8%)	33 (20%)	675 (20%)	353 (52%)				
Employment												
Working	2336 (21%)	592 (25%)	2573 (44%)	824 (32%)	1323 (65%)	271 (21%)	504 (15%)	217 (43%)	1320 (40%)	214 (16%)	657 (18%)	256 (39%)
Not working	4431 (40%)	2119 (48%)	2484 (43%)	1413 (57%)	539 (26%)	258 (48%)	1141 (33%)	712 (62%)	869 (26%)	332 (38%)	2653 (71%)	1862 (70%)
Retired/too old to work	4279 (39%)	835 (20%)	759 (13%)	141 (19%)	183 (9%)	21 (12%)	1796 (52%)	719 (40%)	1110 (34%)	139 (13%)	442 (12%)	111 (25%)
Household wealth												
1st (high) quintile	2169 (20%)	776 (36%)	960 (17%)	462 (48%)	442 (22%)	126 (29%)	629 (18%)	226 (36%)	596 (18%)	153 (26%)	750 (20%)	491 (66%)
2nd	2231 (20%)	878 (39%)	1087 (19%)	509 (47%)	436 (21%)	104 (24%)	694 (20%)	286 (41%)	655 (20%)	151 (23%)	743 (20%)	469 (63%)
3rd	2236 (20%)	751 (34%)	1062 (18%)	449 (42%)	363 (18%)	98 (27%)	690 (20%)	329 (48%)	657 (20%)	128 (20%)	750 (20%)	500 (67%)
4th	2292 (21%)	702 (31%)	1252 (22%)	460 (37%)	417 (20%)	114 (27%)	690 (20%)	392 (57%)	689 (21%)	120 (17%)	773 (21%)	433 (56%)
5th (low) quintile	2118 (19%)	439 (21%)	1455 (25%)	498 (34%)	386 (19%)	108 (28%)	738 (21%)	415 (56%)	702 (21%)	133 (19%)	736 (20%)	336 (46%)
**Health and Health-Risk Factors**												
Body mass index												
Underweight	506 (5%)	151 (30%)	2025 (35%)	814 (40%)	^d^		^d^		^d^		581 (16%)	331 (57%)
Normal weight	6896 (62%)	2382 (35%)	2953 (51%)	1251 (42%)							2126 (57%)	1368 (64%)
Overweight	3060 (28%)	872 (29%)	666 (12%)	260 (39%)							700 (19%)	368 (53%)
Obese	584 (5%)	141 (24%)	172 (3%)	53 (31%)							345 (9%)	162 (47%)
Alcohol use												
Never drunk	7664 (69%)	2152 (28%)	4885 (84%)	1950 (40%)	1066 (52%)	251 (24%)	915 (27%)	345 (38%)	2396 (73%)	490 (21%)	1535 (41%)	858 (56%)
Drunk in the past	1274 (12%)	477 (37%)	551 (10%)	233 (42%)	755 (37%)	227 (30%)	1738 (51%)	835 (48%)	469 (14%)	113 (24%)	1176 (31%)	674 (57%)
≤1 drink per day	849 (8%)	318 (38%)	293 (5%)	145 (50%)	182 (9%)	53 (29%)	626 (18%)	370 (59%)	252 (8%)	46 (18%)	564 (15%)	352 (62%)
>1 drink per day	1259 (11%)	599 (48%)	87 (2%)	50 (58%)	42 (2%)	19 (45%)	162 (5%)	98 (61%)	179 (5%)	36 (20%)	477 (13%)	345 (72%)
Smoking and tobacco use												
No	8032 (73%)	2339 (29%)	3018 (52%)	1120 (37%)	1670 (82%)	443 (27%)	2828 (82%)	1311 (46%)	2429 (74%)	538 (22%)	3280 (87%)	1904 (58%)
Less than daily	282 (3%)	97 (34%)	167 (3%)	48 (29%)	122 (6%)	35 (29%)	49 (1%)	18 (37%)	131 (4%)	14 (11%)	106 (3%)	76 (72%)
Daily	2732 (25%)	1110 (41%)	2631 (45%)	1210 (46%)	253 (12%)	72 (29%)	559 (16%)	316 (57%)	739 (22%)	133 (18%)	366 (10%)	249 (68%)
Non-communicable diseases												
0 diseases	5305 (48%)	2030 (38%)	3042 (52%)	1391 (46%)	776 (38%)	249 (32%)	825 (24%)	439 (53%)	1607 (49%)	414 (26%)	2526 (67%)	1609 (64%)
1 disease	3281 (30%)	1007 (31%)	1663 (29%)	656 (39%)	728 (36%)	187 (26%)	818 (24%)	450 (55%)	953 (29%)	187 (20%)	873 (23%)	472 (54%)
2 diseases	1585 (14%)	356 (23%)	733 (13%)	237 (32%)	368 (18%)	87 (24%)	805 (23%)	387 (48%)	465 (14%)	58 (13%)	271 (7%)	114 (42%)
3+ diseases	875 (8%)	153 (18%)	378 (7%)	94 (25%)	173 (9%)	27 (16%)	993 (29%)	372 (38%)	274 (8%)	26 (10%)	82 (2%)	34 (42%)
Pain												
None	5773 (52%)	1792 (31%)	1585 (27%)	715 (45%)	866 (42%)	269 (31%)	1208 (35%)	650 (54%)	1205 (37%)	351 (29%)	725 (19%)	387 (53%)
Mild	3632 (33%)	1233 (34%)	1971 (34%)	806 (41%)	566 (28%)	169 (30%)	996 (29%)	486 (49%)	966 (29%)	187 (19%)	1356 (36%)	929 (69%)
Moderate	1342 (12%)	461 (34%)	1278 (22%)	500 (39%)	427 (21%)	83 (19%)	795 (23%)	354 (45%)	785 (24%)	101 (13%)	1108 (30%)	641 (58%)
Severe/extreme	299 (3%)	60 (20%)	982 (17%)	357 (36%)	186 (9%)	29 (16%)	438 (13%)	156 (36%)	343 (10%)	46 (13%)	563 (15%)	272 (48%)
Self-rated health												
Good/very good	3755 (34%)	1359 (36%)	1685 (29%)	834 (50%)	760 (37%)	245 (32%)	442 (13%)	261 (59%)	1292 (39%)	401 (31%)	1530 (41%)	1044 (68%)
Moderate	5047 (46%)	1527 (30%)	2990 (51%)	1188 (40%)	1024 (50%)	252 (25%)	2044 (59%)	1100 (54%)	1483 (45%)	228 (15%)	1616 (43%)	950 (59%)
Bad/very bad	2244 (20%)	660 (29%)	1141 (20%)	356 (31%)	261 (13%)	53 (20%)	955 (28%)	287 (30%)	524 (16%)	56 (11%)	606 (16%)	235 (39%)
**Functional Factors**												
Mobility												
Mobility	10451 (95%)	3468 (33%)	5087 (88%)	2191 (43%)	1502 (73%)	474 (32%)	^d^		^d^		2379 (64%)	1492 (63%)
Dismobility	595 (5%)	78 (13%)	729 (13%)	187 (26%)	543 (27%)	76 (14%)					1315 (36%)	712 (54%)
Distance vision impairment												
Mild or none	9847 (89%)	3272 (33%)	4832 (83%)	2086 (43%)	^d^		^d^		2901 (88%)	632 (22%)	3296 (88%)	1971 (60%)
Moderate or greater	1199 (11%)	274 (23%)	984 (17%)	292 (30%)					398 (12%)	53 (13%)	456 (12%)	258 (57%)
Near vision impairment												
Mild or none	7110 (64%)	2321 (33%)	3346 (58%)	1419 (42%)	^d^		^d^		2140 (65%)	455 (21%)	2730 (73%)	1665 (61%)
Moderate or greater	3936 (36%)	1225 (31%)	2458 (42%)	955 (39%)					1159 (35%)	230 (20%)	1021 (27%)	563 (55%)
	**Mean (SD)**	**Mean (SD)**	**Mean (SD)**	**Mean (SD)**	**Mean (SD)**	**Mean (SD)**	**Mean (SD)**	**Mean (SD)**	**Mean (SD)**	**Mean (SD)**	**Mean (SD)**	**Mean (SD)**
Verbal learning and memory												
Words recalled immediately ^e^	5.7 (1.6)	5.6 (1.6)	5.4 (1.4)	5.5 (1.3)	5.2 (1.4)	5.4 (1.3)	6.0 (1.6)	6.4 (1.5)	5.9 (1.6)	6.4 (1.3)	5.8 (1.4)	6.0 (1.4)
Words lost with delay ^e^	1.6 (1.6)	1.7 (1.6)	1.8 (1.5)	1.8 (1.5)	2.3 (2.0)	2.4 (2.1)	1.9 (1.5)	1.9 (1.5)	^d^		1.8 (1.6)	1.8 (1.7)
IADL ^e^	0.21 (0.48)	0.11 (0.29)	0.94 (0.86)	0.77 (0.73)	0.65 (0.89)	0.32 (0.51)	0.66 (0.88)	0.38 (0.57)	0.54 (0.84)	0.21 (0.50)	0.72 (0.79)	0.58 (0.61)
ADL ^e^	0.23 (0.40)	0.15 (0.26)	0.87 (0.69)	0.71 (0.58)	0.76 (0.76)	0.47 (0.49)	0.68 (0.73)	0.45 (0.51)	0.62 (0.72)	0.34 (0.49)	0.73 (0.67)	0.61 (0.54)
Disability ^e^	7.3 (10.8)	5.6 (7.7)	23.6 (17.7)	20.1 (15.1)	16.5 (17.3)	10.5 (10.7)	18.6 (17.4)	13.4 (12.3)	16.5 (18.0)	9.5 (11.9)	19.5 (17.2)	16.6 (14.6)
Quality of Life ^e^	65.1 (14.2)	66.1 (13.1)	61.2 (14.8)	63.0 (14.1)	65.7 (12.9)	68.4 (11.7)	59.8 (15.3)	62.5 (13.8)	60.1 (15.2)	63.2 (14.6)	56.7 (15.4)	58.2 (14.1)
**Interpersonal Factors**												
Emotional loneliness												
No	10364 (94%)	3361 (32%)	4883 (84%)	2030 (42%)	1681 (82%)	473 (28%)	2973 (86%)	1482 (50%)	2977 (91%)	629 (21%)	3370 (90%)	2050 (61%)
Yes	623 (6%)	167 (27%)	933 (16%)	348 (37%)	364 (18%)	77 (21%)	468 (14%)	166 (36%)	310 (9%)	53 (17%)	382 (10%)	179 (47%)
Friends visiting home												
Never	2935 (27%)	884 (30%)	1050 (18%)	396 (38%)	963 (47%)	216 (22%)	469 (14%)	161 (34%)	418 (13%)	53 (13%)	433 (12%)	149 (34%)
Once or twice per year	4800 (44%)	1409 (29%)	1342 (23%)	514 (38%)	417 (20%)	117 (28%)	1408 (41%)	697 (50%)	410 (12%)	96 (23%)	365 (10%)	153 (42%)
Once or twice per month	2481 (23%)	896 (36%)	1718 (30%)	728 (42%)	258 (13%)	78 (30%)	1192 (35%)	588 (49%)	979 (30%)	204 (21%)	774 (21%)	380 (49%)
Once per week or more	830 (8%)	357 (43%)	1706 (29%)	740 (43%)	407 (20%)	139 (34%)	372 (11%)	202 (54%)	1492 (45%)	332 (22%)	2180 (58%)	1547 (71%)
Visiting people ^f^												
Never	3902 (35%)	1101 (28%)	924 (16%)	333 (36%)	1063 (52%)	259 (24%)	1054 (31%)	404 (38%)	567 (17%)	74 (13%)	640 (17%)	221 (35%)
Once or twice per year	4800 (44%)	1559 (33%)	1639 (28%)	666 (41%)	468 (23%)	142 (30%)	1504 (44%)	762 (51%)	608 (18%)	157 (26%)	506 (14%)	229 (45%)
Once or twice per month	1598 (15%)	570 (36%)	1613 (28%)	653 (41%)	264 (13%)	80 (30%)	721 (21%)	387 (54%)	1085 (33%)	191 (18%)	935 (25%)	535 (57%)
Once per week or more	746 (7%)	316 (42%)	1638 (28%)	726 (44%)	250 (12%)	69 (28%)	148 (4%)	86 (58%)	1039 (32%)	263 (25%)	1671 (45%)	1244 (74%)
Socializing with coworkers												
Never	3084 (28%)	850 (28%)	2380 (41%)	815 (34%)	1503 (74%)	352 (23%)	1672 (49%)	624 (37%)	^d^		1551 (41%)	671 (43%)
Once or twice per year	2773 (25%)	764 (28%)	1338 (23%)	527 (39%)	279 (14%)	94 (34%)	806 (23%)	441 (55%)			378 (10%)	203 (54%)
Once or twice per month	2466 (22%)	764 (31%)	1045 (18%)	450 (43%)	139 (7%)	45 (32%)	552 (16%)	321 (58%)			575 (15%)	396 (69%)
Once per week or more	2723 (25%)	1168 (43%)	1053 (18%)	586 (56%)	124 (6%)	59 (48%)	411 (12%)	262 (64%)			1248 (33%)	959 (77%)
Social activities outside of home												
Never	2080 (19%)	495 (24%)	1231 (21%)	392 (32%)	957 (47%)	223 (23%)	1260 (37%)	496 (39%)	774 (24%)	120 (16%)	483 (13%)	188 (39%)
Once or twice per year	6567 (60%)	2201 (34%)	3215 (55%)	1355 (42%)	631 (31%)	196 (31%)	1002 (29%)	475 (47%)	741 (23%)	196 (27%)	647 (17%)	335 (52%)
Once or twice per month	1956 (18%)	680 (35%)	1088 (19%)	496 (46%)	288 (14%)	81 (28%)	702 (20%)	387 (55%)	999 (30%)	233 (23%)	953 (25%)	559 (59%)
Once per week or more	443 (4%)	170 (38%)	282 (5%)	135 (48%)	169 (8%)	50 (30%)	477 (14%)	290 (61%)	785 (24%)	136 (17%)	1669 (45%)	1147 (69%)
Marital status												
Coupled	9235 (84%)	3070 (33%)	4398 (76%)	1890 (43%)	1246 (61%)	385 (31%)	1948 (57%)	1037 (53%)	1750 (53%)	406 (23%)	2139 (57%)	1401 (66%)
Uncoupled	1811 (16%)	476 (26%)	1418 (24%)	488 (34%)	799 (39%)	165 (21%)	1493 (43%)	611 (41%)	1549 (47%)	279 (18%)	1613 (43%)	828 (51%)
**Environmental Factors**												
Location												
Urban	5230 (47%)	1135 (22%)	1444 (25%)	478 (33%)	1470 (72%)	378 (26%)	2609 (76%)	1190 (46%)	2212 (67%)	439 (20%)	1501 (40%)	685 (46%)
Rural	5816 (53%)	2411 (42%)	4372 (75%)	1900 (44%)	575 (28%)	172 (30%)	832 (24%)	458 (55%)	1086 (33%)	246 (23%)	2251 (60%)	1544 (69%)
Personal motorized transport												
No	5014 (45%)	1735 (35%)	4291 (74%)	1859 (43%)	1300 (64%)	356 (27%)	1965 (57%)	845 (43%)	2428 (74%)	518 (21%)	3551 (95%)	2153 (61%)
Yes	6032 (55%)	1811 (30%)	1525 (26%)	519 (34%)	744 (36%)	194 (26%)	1476 (43%)	803 (54%)	871 (26%)	167 (19%)	201 (5%)	76 (38%)
Computer in household												
No	8585 (78%)	3056 (36%)	5525 (95%)	2283 (41%)	1730 (85%)	478 (28%)	2782 (81%)	1294 (47%)	2827 (86%)	585 (21%)	3577 (95%)	2168 (61%)
Yes	2461 (22%)	490 (20%)	291 (5%)	95 (33%)	314 (15%)	72 (23%)	659 (19%)	354 (54%)	455 (14%)	98 (22%)	175 (5%)	61 (35%)
Safety out on the street after dark												
Completely/very safe	6635 (60%)	2340 (35%)	3549 (61%)	1521 (43%)	722 (35%)	199 (28%)	535 (16%)	261 (49%)	491 (15%)	119 (24%)	2751 (73%)	1696 (62%)
Moderately safe	2872 (26%)	704 (25%)	1400 (24%)	523 (37%)	498 (24%)	133 (27%)	995 (29%)	497 (50%)	635 (19%)	136 (21%)	694 (19%)	405 (58%)
Slightly safe/not at all	1539 (14%)	502 (33%)	867 (15%)	334 (39%)	825 (40%)	218 (26%)	1865 (55%)	875 (47%)	2149 (66%)	427 (20%)	307 (8%)	128 (42%)
Safety when home alone												
Completely/very safe	9283 (84%)	3012 (32%)	3924 (68%)	1668 (43%)	1133 (55%)	320 (28%)	1485 (43%)	740 (50%)	785 (24%)	214 (27%)	3186 (85%)	1996 (63%)
Moderately safe	1431 (13%)	409 (29%)	1364 (24%)	519 (38%)	422 (21%)	104 (25%)	1123 (33%)	507 (45%)	904 (27%)	169 (19%)	400 (11%)	179 (45%)
Slightly safe/not at all	330 (3%)	123 (37%)	528 (9%)	191 (36%)	490 (24%)	126 (26%)	823 (24%)	393 (48%)	1610 (49%)	302 (19%)	166 (4%)	54 (33%)

^a^ Distribution of the factor in each country’s sample. ^b^ Percentage of people meeting physical activity guidelines (active) for each factor level. ^c^ Education levels for some countries collapsed (e.g., secondary/high and college or more for India) due to low frequencies. ^d^ Factor omitted from the analyses due to large amounts of missing or implausible data. ^e^ Range: 0–10 for words recalled immediately (higher scores = higher function) and words lost with delay (higher scores = lower function), 0–5 for instrumental activities of daily living (IADL) and activities of daily living (ADL) (higher scores = lower function), and 0–100 for disability (higher scores = lower function) and quality of life (higher scores = higher function). ^f^ Visiting people in different neighborhoods or them coming to visit.

**Table 2:** Multivariate associations of meeting physical activity guidelines with demographic, health and health risk, functional, interpersonal, and environmental factors.

Factors	China		India		Mexico		Russian Federation		South Africa		Ghana	
	OR (95% CI) ^a^	*p* ^a^	OR (95% CI) ^a^	*p* ^a^	OR (95% CI) ^a^	*p* ^a^	OR (95% CI) ^a^	*p* ^a^	OR (95% CI) ^a^	*p* ^a^	OR (95% CI) ^a^	*p* ^a^
**Demographic Factors**												
Age (Ref: 50–59)												
60–69	0.79 (0.71, 0.88)	<0.0001	0.76 (0.67, 0.87)	<0.0001	0.99 (0.76, 1.31)	0.0347	0.74 (0.60, 0.90)	<0.0001	0.69 (0.54, 0.86)	0.0008	0.79 (0.64, 0.97)	<0.0001
70–79	0.60 (0.52, 0.70)		0.50 (0.41, 0.61)		0.82 (0.59, 1.14)		0.54 (0.42, 0.68)		0.60 (0.43, 0.83)		0.50 (0.40, 0.64)	
80+	0.38 (0.27, 0.52)		0.28 (0.18, 0.44)		0.48 (0.27, 0.82)		0.28 (0.19, 0.40)		0.49 (0.28, 0.82)		0.43 (0.31, 0.60)	
Sex (Ref: Male)												
Female	0.97 (0.86, 1.10)	0.6709	1.58 (1.34, 1.87)	<0.0001	1.13 (0.86, 1.50)	0.3933	1.23 (1.03, 1.46)	0.0199	0.83 (0.68, 1.02)	0.0755	0.80 (0.65, 0.99)	0.0408
Education completed (Ref: Lowest level, country-specific) ^b^												
Less than primary school	1.02 (0.89, 1.17)	<0.0001	1.22 (1.00, 1.48)	0.0003	0.97 (0.72, 1.30)	0.1669			^c^			
Primary school	1.08 (0.94, 1.25)		1.48 (1.24, 1.78)		0.89 (0.63, 1.26)						0.72 (0.55, 0.95)	<0.0001
Secondary/high school	0.75 (0.65, 0.87)		1.21 (1.01, 1.46)		0.90 (0.58, 1.38)		1.24 (0.94, 1.63)	0.0797			0.53 (0.42, 0.66)	
College or more	0.38 (0.27, 0.52)				0.54 (0.33, 0.89)		1.04 (0.76, 1.44)					
Employment (Ref: Working)												
Not working	0.52 (0.46, 0.59)	<0.0001	0.45 (0.39, 0.52)	<0.0001	0.39 (0.30, 0.51)	<0.0001	0.86 (0.67, 1.10)	0.4431	0.47 (0.38, 0.59)	<0.0001	0.49 (0.39, 0.61)	<0.0001
Retired/too old to work	0.56 (0.49, 0.64)		0.29 (0.23, 0.36)		0.23 (0.13, 0.38)		0.91 (0.73, 1.13)		0.46 (0.35, 0.61)		0.32 (0.24, 0.42)	
Household wealth (Ref: 1st (high) quintile)												
2nd	1.32 (1.12, 1.56)	<0.0001	0.95 (0.78, 1.15)	<0.0001	^d^		1.05 (0.82, 1.33)	0.0007	1.27 (0.94, 1.71)	<0.0001	0.91 (0.70, 1.19)	0.0055
3rd	1.50 (1.25, 1.81)		1.19 (0.95, 1.48)				0.90 (0.69, 1.17)		1.41 (1.04, 1.91)		1.34 (1.00, 1.78)	
4th	1.80 (1.47, 2.20)		1.51 (1.20, 1.90)				0.70 (0.53, 0.93)		1.71 (1.27, 2.32)		1.19 (0.88, 1.59)	
5th (low) quintile	1.76 (1.41, 2.20)		1.67 (1.31, 2.13)				0.62 (0.46, 0.85)		2.23 (1.64, 3.04)		1.48 (1.08, 2.02)	
**Health and Health-Risk Factors**												
Body mass index (Ref: Normal weight)												
Underweight	0.87 (0.70, 1.08)	0.0060	0.94 (0.82, 1.07)	0.3851	^c^		^c^		^c^		0.95 (0.75, 1.20)	0.0053
Overweight	0.86 (0.78, 0.96)		1.04 (0.86, 1.26)								0.67 (0.54, 0.84)	
Obese	0.78 (0.63, 0.96)		0.79 (0.55, 1.12)								0.81 (0.60, 1.09)	
Alcohol use (Ref: Never drunk)												
Drunk in the past	1.53 (1.33, 1.77)	<0.0001	1.07 (0.86, 1.31)	0.8356	1.13 (0.88, 1.46)	0.4463	1.46 (1.22, 1.75)	<0.0001	^d^		1.18 (0.97, 1.44)	0.3704
≤1 drink per day	1.17 (0.99, 1.38)		1.11 (0.85, 1.45)		0.86 (0.57, 1.29)		1.76 (1.39, 2.23)				1.04 (0.81, 1.33)	
>1 drink per day	1.59 (1.37, 1.85)		1.08 (0.68, 1.74)		1.28 (0.62, 2.61)		1.83 (1.25, 2.68)				1.14 (0.86, 1.52)	
Smoking and tobacco use (Ref: No)												
Less than daily	0.91 (0.68, 1.20)	0.7531	0.66 (0.45, 0.95)	0.0002	^d^		^d^		0.40 (0.21, 0.71)	0.0011	1.43 (0.87, 2.41)	0.3737
Daily	1.01 (0.89, 1.14)		1.22 (1.07, 1.38)						0.75 (0.59, 0.94)		1.07 (0.79, 1.44)	
Non-communicable diseases (Ref: 0 diseases)												
1 disease	0.87 (0.78, 0.96)	<0.0001	0.98 (0.85, 1.12)	0.1466	0.91 (0.71, 1.16)	0.7920	1.29 (1.04, 1.60)	0.0723	0.93 (0.75, 1.16)	0.1304	1.01 (0.83, 1.23)	0.2363
2 diseases	0.73 (0.63, 0.84)		0.86 (0.71, 1.04)		0.97 (0.70, 1.32)		1.22 (0.98, 1.53)		0.81 (0.58, 1.12)		0.78 (0.57, 1.07)	
3+ diseases	0.70 (0.56, 0.86)		0.77 (0.58, 1.01)		0.82 (0.50, 1.32)		1.08 (0.86, 1.37)		0.60 (0.37, 0.93)		1.41 (0.80, 2.46)	
Pain (Ref: None)												
Mild	1.35 (1.22, 1.50)	<0.0001	^d^		1.14 (0.88, 1.48)	0.3482	^d^		1.01 (0.80, 1.27)	0.0171	2.53 (2.00, 3.20)	<0.0001
Moderate	1.68 (1.44, 1.97)				0.86 (0.61, 1.19)				1.13 (0.83, 1.55)		2.12 (1.64, 2.75)	
Severe/extreme	1.10 (0.79, 1.53)				0.88 (0.53, 1.42)				2.00 (1.27, 3.12)		1.96 (1.42, 2.70)	
Self-rated health (Ref: Good/very good)												
Moderate	0.88 (0.79, 0.98)	0.0668	0.85 (0.74, 0.97)	0.0398	0.83 (0.66, 1.06)	0.1812	0.94 (0.74, 1.18)	<0.0001	0.63 (0.50, 0.80)	0.0004	0.82 (0.67, 0.99)	<0.0001
Bad/very bad	0.93 (0.80, 1.09)		0.81 (0.67, 0.99)		1.09 (0.72, 1.63)		0.53 (0.40, 0.71)		0.62 (0.42, 0.92)		0.49 (0.38, 0.65)	
**Functional Factors**												
Mobility (Ref: Mobility)												
Dismobility	0.65 (0.49, 0.85)	0.0018	0.81 (0.66, 0.99)	0.0421	0.76 (0.55, 1.04)	0.0851	^c^		^c^		1.22 (0.95, 1.58)	0.1289
Distance vision impairment (Ref: Mild or none)												
Moderate or greater	0.83 (0.71, 0.98)	0.0280	0.81 (0.68, 0.95)	0.0110	^c^		^c^		0.81 (0.58, 1.12)	0.2128	^d^	
Near vision impairment (Ref: Mild or none)												
Moderate or greater	1.03 (0.94, 1.14)	0.5424	^d^		^c^		^c^		^d^		^d^	
Verbal learning and memory												
Words recalled immediately ^e^	^d^		1.05 (1.00, 1.10)	0.0393	^d^		^d^		1.13 (1.05, 1.21)	0.0005	1.15 (1.08, 1.23)	<0.0001
Words lost with delay ^e^	^d^		^d^		^d^		^d^		^c^		1.01 (0.96, 1.06)	0.8200
IADL ^e^	0.46 (0.39, 0.54)	<0.0001	0.85 (0.78, 0.92)	0.0002	0.59 (0.48, 0.73)	<0.0001	^d^		0.53 (0.42, 0.65)	<0.0001	0.79 (0.69, 0.91)	0.0010
Quality of Life ^e^	1.00 (1.00, 1.01)	0.5159	^d^		^d^		^d^		^d^		^d^	
**Interpersonal Factors**												
Emotional loneliness (Ref: No)												
Yes	^d^		0.98 (0.83, 1.16)	0.8249	0.81 (0.60, 1.10)	0.1796	0.99 (0.79, 1.26)	0.9576	^d^		1.18 (0.90, 1.54)	0.2329
Friends visiting home (Ref: Never)												
Once or twice per year	0.80 (0.71, 0.91)	0.0005	^d^		1.24 (0.89, 1.73)	0.0074	1.21 (0.95, 1.54)	0.0019	1.21 (0.79, 1.87)	0.1728	0.84 (0.57, 1.22)	<0.0001
Once or twice per month	0.91 (0.79, 1.05)				1.58 (1.08, 2.30)		0.86 (0.67, 1.12)		1.41 (0.95, 2.11)		1.10 (0.79, 1.54)	
Once per week or more	1.07 (0.88, 1.29)				1.78 (1.26, 2.52)		1.09 (0.79, 1.49)		1.56 (1.04, 2.35)		2.26 (1.65, 3.11)	
Visiting people (Ref: Never) ^f^												
Once or twice per year	1.00 (0.89, 1.13)	0.8431	^d^		0.95 (0.69, 1.30)	0.0187	1.39 (1.15, 1.69)	0.0007	1.33 (0.92, 1.91)	0.0001	1.25 (0.91, 1.72)	<0.0001
Once or twice per month	1.03 (0.88, 1.20)				0.73 (0.50, 1.07)		1.36 (1.08, 1.71)		0.72 (0.50, 1.03)		1.20 (0.89, 1.61)	
Once per week or more	1.08 (0.89, 1.31)				0.53 (0.35, 0.80)		1.55 (1.19, 2.03)		1.15 (0.79, 1.68)		1.97 (1.47, 2.65)	
Socializing with coworkers (Ref: Never)												
Once or twice per year	1.03 (0.90, 1.19)	<0.0001	1.02 (0.87, 1.19)	<0.0001	1.33 (0.95, 1.87)	0.0514	^d^		^c^		1.31 (0.98, 1.75)	<0.0001
Once or twice per month	1.02 (0.88, 1.17)		1.05 (0.88, 1.25)		1.00 (0.65, 1.53)						2.14 (1.66, 2.75)	
Once per week or more	1.32 (1.15, 1.51)		1.53 (1.29, 1.81)		1.72 (1.10, 2.68)						2.10 (1.69, 2.61)	
Social activities outside of home (Ref: Never)												
Once or twice per year	1.32 (1.16, 1.51)	<0.0001	1.28 (1.09, 1.49)	0.0042	^d^		1.12 (0.93, 1.35)	<0.0001	1.26 (0.94, 1.69)	0.0139	1.08 (0.79, 1.48)	0.5466
Once or twice per month	1.52 (1.30, 1.78)		1.41 (1.16, 1.71)				1.37 (1.11, 1.69)		1.10 (0.84, 1.46)		1.23 (0.91, 1.67)	
Once per week or more	1.63 (1.28, 2.08)		1.27 (0.95, 1.70)				1.80 (1.41, 2.30)		0.78 (0.57, 1.07)		1.11 (0.83, 1.48)	
Marital status (Ref: Coupled)												
Uncoupled	1.03 (0.90, 1.17)	0.6973	1.02 (0.88, 1.18)	0.8269	0.75 (0.58, 0.95)	0.0195	0.94 (0.80, 1.12)	0.5125	0.98 (0.79, 1.20)	0.8305	0.80 (0.65, 0.98)	0.0337
**Environmental Factors**												
Location (Ref: Urban)												
Rural	1.16 (1.01, 1.32)	0.0323	1.39 (1.20, 1.61)	<0.0001	1.17 (0.91, 1.50)	0.2114	1.37 (1.15, 1.65)	0.0006	^d^		1.71 (1.42, 2.06)	<0.0001
Personal motorized transport (Ref: No)												
Yes	0.99 (0.88, 1.10)	0.7874	0.81 (0.68, 0.96)	0.0151	^d^		0.98 (0.82, 1.18)	0.8555	^d^		0.58 (0.39, 0.84)	0.0046
Computer in household (Ref: No)												
Yes	0.75 (0.64, 0.88)	0.0004	^d^		^d^		0.75 (0.60, 0.94)	0.0121	^d^		0.60 (0.39, 0.90)	0.0149
Safety out on the street after dark (Ref: Completely/very safe)												
Moderately safe	0.78 (0.70, 0.86)	<0.0001	^d^		^d^		^d^		^d^		1.48 (1.16, 1.89)	0.0004
Slightly safe/not at all	1.20 (1.05, 1.37)										0.77 (0.54, 1.10)	
Safety when home alone (Ref: Completely/very safe)												
Moderately safe	^d^		0.83 (0.72, 0.95)	0.0033	^d^		^d^		0.70 (0.54, 0.90)	0.0022	0.56 (0.41, 0.76)	<0.0001
Slightly safe/not at all			0.77 (0.62, 0.94)						0.68 (0.54, 0.85)		0.45 (0.28, 0.70)	

^a^ Odds ratios (95% confidence intervals) and p values for those factors that were selected in the fully adjusted final model for each country. ^b^ Education levels for some countries collapsed (e.g., secondary/high and college or more for India) due to low numbers. ^c^ Factor omitted from the analyses due to large amounts of missing or implausible data. ^d^ Factor was not selected for the final model. ^e^ Higher scores = higher function for words recalled immediately and quality of life; higher scores = lower function for words lost with delay and IADL. ^f^ Visiting people in different neighborhoods or them coming to visit.

**Table 3:** Descriptive statistics for the samples of adults and for those who were sedentary ≥4 h/day.

Factors	China		India		Mexico		Russian Federation		South Africa		Ghana	
	Sample	Sedentary	Sample	Sedentary	Sample	Sedentary	Sample	Sedentary	Sample	Sedentary	Sample	Sedentary
	n (%) ^a^	n (%) ^b^	n (%) ^a^	n (%) ^b^	n (%) ^a^	n (%) ^b^	n (%) ^a^	n (%) ^b^	n (%) ^a^	n (%) ^b^	n (%) ^a^	n (%) ^b^
**Demographic Factors**												
Age												
50–59	5082 (45%)	1953 (38%)	2806 (46%)	888 (32%)	396 (19%)	58 (15%)	1317 (38%)	616 (47%)	1472 (44%)	467 (32%)	1561 (40%)	559 (36%)
60–69	3415 (30%)	1533 (45%)	2100 (34%)	800 (38%)	850 (41%)	148 (17%)	942 (27%)	552 (59%)	1087 (32%)	411 (38%)	1098 (28%)	449 (41%)
70–79	2306 (20%)	1242 (54%)	982 (16%)	476 (49%)	565 (28%)	133 (24%)	860 (25%)	559 (65%)	594 (18%)	265 (45%)	869 (22%)	449 (52%)
80+	552 (5%)	357 (65%)	283 (5%)	161 (57%)	241 (12%)	93 (39%)	327 (10%)	270 (83%)	209 (6%)	97 (46%)	372 (10%)	228 (61%)
Sex												
Male	5260 (46%)	2236 (43%)	3153 (51%)	1211 (38%)	798 (39%)	183 (23%)	1230 (36%)	690 (56%)	1438 (43%)	512 (36%)	2040 (52%)	789 (39%)
Female	6095 (54%)	2849 (47%)	3018 (49%)	1114 (37%)	1254 (61%)	249 (20%)	2216 (64%)	1307 (59%)	1924 (57%)	728 (38%)	1860 (48%)	896 (48%)
Education completed ^c^												
Never been to school	2766 (24%)	1193 (43%)	3144 (51%)	1160 (37%)	411 (20%)	87 (21%)			^d^			
Less than primary school	2117 (19%)	845 (40%)	698 (11%)	267 (38%)	849 (41%)	177 (21%)					2526 (65%)	1134 (45%)
Primary school	2320 (20%)	988 (43%)	881 (14%)	360 (41%)	438 (21%)	77 (18%)	367 (11%)	270 (74%)			419 (11%)	181 (43%)
Secondary/high school	3672 (32%)	1763 (48%)	1448 (24%)	538 (37%)	186 (9%)	50 (27%)	2399 (70%)	1338 (56%)			955 (25%)	370 (39%)
College or more	480 (4%)	296 (62%)			168 (8%)	41 (24%)	680 (20%)	389 (57%)				
Employment												
Working	2396 (21%)	1149 (48%)	2753 (45%)	1182 (43%)	1329 (65%)	279 (21%)	493 (14%)	311 (63%)	1363 (41%)	553 (41%)	676 (17%)	400 (59%)
Not working	4583 (40%)	1547 (34%)	2583 (42%)	726 (28%)	543 (27%)	93 (17%)	1158 (34%)	531 (46%)	887 (26%)	223 (25%)	2770 (71%)	983 (36%)
Retired/too old to work	4376 (39%)	2389 (55%)	835 (14%)	417 (50%)	180 (9%)	60 (33%)	1795 (52%)	1155 (64%)	1112 (33%)	464 (42%)	454 (12%)	302 (67%)
Household wealth												
1st (high) quintile	2229 (20%)	821 (37%)	1016 (17%)	403 (40%)	442 (22%)	83 (19%)	624 (18%)	401 (64%)	614 (18%)	247 (40%)	781 (20%)	351 (45%)
2nd	2295 (20%)	896 (39%)	1157 (19%)	469 (41%)	439 (21%)	92 (21%)	694 (20%)	431 (62%)	663 (20%)	294 (44%)	760 (20%)	330 (43%)
3rd	2302 (20%)	1018 (44%)	1141 (19%)	427 (37%)	365 (18%)	80 (22%)	689 (20%)	394 (57%)	661 (20%)	240 (36%)	775 (20%)	301 (39%)
4th	2345 (21%)	1185 (51%)	1330 (22%)	437 (33%)	418 (20%)	99 (24%)	694 (20%)	378 (55%)	705 (21%)	235 (33%)	799 (21%)	347 (43%)
5th (low) quintile	2184 (19%)	1165 (53%)	1527 (25%)	589 (39%)	387 (19%)	77 (20%)	745 (22%)	393 (53%)	719 (21%)	224 (31%)	785 (20%)	356 (45%)
**Health and Health-Risk Factors**												
Body mass index												
Underweight	515 (5%)	231 (45%)	2181 (35%)	893 (41%)	^d^		^d^		^d^		600 (15%)	276 (46%)
Normal weight	7091 (62%)	2971 (42%)	3112 (50%)	1129 (36%)							2204 (57%)	894 (41%)
Overweight	3156 (28%)	1564 (50%)	696 (11%)	239 (34%)							734 (19%)	333 (45%)
Obese	593 (5%)	319 (54%)	182 (3%)	64 (35%)							362 (9%)	182 (50%)
Alcohol use												
Never drunk	7867 (69%)	3657 (47%)	5177 (84%)	1956 (38%)	1069 (52%)	214 (20%)	914 (27%)	497 (54%)	2433 (73%)	883 (36%)	1598 (41%)	684 (43%)
Drunk in the past	1301 (12%)	590 (45%)	592 (10%)	265 (45%)	758 (37%)	178 (24%)	1742 (51%)	1070 (61%)	480 (14%)	175 (37%)	1223 (31%)	597 (49%)
≤1 drink per day	868 (8%)	327 (38%)	311 (5%)	69 (22%)	183 (9%)	35 (19%)	627 (18%)	333 (53%)	257 (8%)	93 (36%)	586 (15%)	227 (39%)
>1 drink per day	1319 (12%)	511 (39%)	91 (2%)	35 (39%)	42 (2%)	5 (12%)	163 (5%)	97 (60%)	188 (6%)	88 (47%)	493 (13%)	177 (36%)
Smoking and tobacco use												
No	8225 (73%)	3817 (46%)	3199 (52%)	1179 (37%)	1676 (82%)	346 (21%)	2833 (82%)	1665 (59%)	2461 (74%)	913 (37%)	3397 (87%)	1498 (44%)
Less than daily	287 (3%)	106 (37%)	187 (3%)	65 (35%)	123 (6%)	27 (22%)	48 (1%)	28 (58%)	130 (4%)	41 (32%)	112 (3%)	37 (33%)
Daily	2811 (25%)	1153 (41%)	2785 (45%)	1081 (39%)	253 (12%)	59 (23%)	560 (16%)	300 (54%)	749 (22%)	280 (37%)	382 (10%)	146 (38%)
Non-communicable diseases												
0 diseases	5446 (48%)	2107 (39%)	3159 (51%)	1085 (34%)	779 (38%)	151 (19%)	833 (24%)	395 (47%)	1644 (49%)	585 (36%)	2616 (67%)	1047 (40%)
1 disease	3366 (30%)	1567 (47%)	1772 (29%)	731 (41%)	729 (36%)	149 (20%)	818 (24%)	428 (52%)	967 (29%)	375 (39%)	916 (24%)	424 (46%)
2 diseases	1646 (15%)	893 (54%)	816 (13%)	331 (41%)	369 (18%)	83 (23%)	811 (24%)	483 (60%)	473 (14%)	184 (39%)	284 (7%)	157 (55%)
3+ diseases	897 (8%)	518 (58%)	424 (7%)	178 (42%)	175 (9%)	49 (28%)	984 (29%)	691 (70%)	278 (8%)	96 (35%)	84 (2%)	57 (68%)
Pain												
None	5917 (52%)	2550 (43%)	1650 (27%)	566 (34%)	874 (43%)	159 (18%)	1223 (36%)	590 (48%)	1232 (37%)	388 (32%)	769 (20%)	267 (35%)
Mild	3744 (33%)	1683 (45%)	2080 (34%)	726 (35%)	567 (28%)	105 (19%)	1002 (29%)	586 (59%)	966 (29%)	365 (38%)	1408 (36%)	574 (41%)
Moderate	1382 (12%)	679 (49%)	1380 (22%)	545 (40%)	427 (21%)	107 (25%)	795 (23%)	525 (66%)	802 (24%)	329 (41%)	1132 (29%)	525 (46%)
Severe/extreme	312 (3%)	173 (55%)	1061 (17%)	488 (46%)	184 (9%)	61 (33%)	426 (12%)	296 (70%)	359 (11%)	158 (44%)	588 (15%)	317 (54%)
Self-rated health												
Good/very good	3851 (34%)	1575 (41%)	1758 (29%)	608 (35%)	763 (37%)	145 (19%)	447 (13%)	193 (43%)	1320 (39%)	410 (31%)	1594 (41%)	491 (31%)
Moderate	5191 (46%)	2285 (44%)	3182 (52%)	1067 (34%)	1026 (50%)	209 (20%)	2064 (60%)	1080 (52%)	1515 (45%)	563 (37%)	1681 (43%)	794 (47%)
Bad/very bad	2313 (20%)	1225 (53%)	1231 (20%)	650 (53%)	263 (13%)	78 (30%)	935 (27%)	724 (77%)	527 (16%)	267 (51%)	625 (16%)	400 (64%)
**Functional Factors**												
Mobility												
Mobility	10745 (95%)	4706 (44%)	5357 (87%)	1895 (35%)	1513 (74%)	255 (17%)	^d^		^d^		2513 (64%)	947 (38%)
Dismobility	610 (5%)	379 (62%)	814 (13%)	430 (53%)	539 (26%)	177 (33%)					1387 (36%)	738 (53%)
Distance vision impairment												
Mild or none	10054 (89%)	4422 (44%)	4888 (83%)	1783 (37%)	^d^		^d^		2961 (88%)	1049 (35%)	3427 (88%)	1426 (42%)
Moderate or greater	1218 (11%)	627 (52%)	997 (17%)	410 (41%)					401 (12%)	191 (48%)	473 (12%)	259 (55%)
Near vision impairment												
Mild or none	7299 (64%)	3119 (43%)	3402 (57%)	1265 (37%)	^d^		^d^		2196 (65%)	797 (36%)	2837 (73%)	1147 (40%)
Moderate or greater	4056 (36%)	1966 (49%)	2521 (43%)	942 (37%)					1166 (35%)	443 (38%)	1063 (27%)	538 (51%)
	**Mean (SD)**	**Mean (SD)**	**Mean (SD)**	**Mean (SD)**	**Mean (SD)**	**Mean (SD)**	**Mean (SD)**	**Mean (SD)**	**Mean (SD)**	**Mean (SD)**	**Mean (SD)**	**Mean (SD)**
Verbal learning and memory												
Words recalled immediately ^e^	5.6 (1.6)	5.7 (1.7)	5.4 (1.4)	5.2 (1.4)	5.2 (1.4)	5.0 (1.5)	6.0 (1.6)	5.8 (1.6)	5.9 (1.6)	5.6 (1.6)	5.8 (1.4)	5.6 (1.4)
Words lost with delay ^e^	1.6 (1.6)	1.7 (1.6)	1.8 (1.5)	1.8 (1.5)	2.3 (2.0)	2.0 (1.9)	1.9 (1.5)	1.9 (1.5)	^d^		1.8 (1.6)	1.9 (1.6)
IADL ^e^	0.21 (0.47)	0.27 (0.56)	0.96 (0.87)	1.13 (0.96)	0.63 (0.88)	1.05 (1.18)	0.64 (0.86)	0.85 (0.96)	0.54 (0.84)	0.72 (0.94)	0.71 (0.78)	0.88 (0.88)
ADL ^e^	0.23 (0.40)	0.28 (0.46)	0.89 (0.69)	1.02 (0.76)	0.75 (0.75)	1.09 (0.95)	0.66 (0.72)	0.84 (0.79)	0.62 (0.72)	0.77 (0.77)	0.73 (0.66)	0.86 (0.72)
Disability ^e^	7.3 (10.6)	8.6 (12.3)	24.0 (17.8)	28.2 (20.0)	16.3 (17.0)	23.8 (21.6)	18.3 (17.0)	22.7 (18.6)	16.5 (17.9)	20.6 (19.2)	19.4 (17.2)	22.8 (18.4)
Quality of Life ^e^	65.1 (14.2)	64.7 (14.9)	61.1 (14.8)	57.7 (15.5)	65.7 (12.8)	62.8 (13.9)	60.1 (15.2)	57.4 (15.6)	60.0 (15.3)	56.0 (15.7)	56.7 (15.5)	53.4 (16.0)
**Interpersonal Factors**												
Emotional loneliness												
No	10645 (94%)	4751 (45%)	5167 (84%)	1926 (37%)	1689 (82%)	333 (20%)	2983 (87%)	1656 (56%)	3037 (90%)	1094 (36%)	3512 (90%)	1420 (40%)
Yes	651 (6%)	305 (47%)	998 (16%)	399 (40%)	363 (18%)	99 (27%)	463 (13%)	341 (74%)	325 (10%)	146 (45%)	388 (10%)	265 (68%)
Friends visiting home												
Never	3024 (27%)	1372 (45%)	1109 (18%)	470 (42%)	962 (47%)	194 (20%)	455 (13%)	312 (69%)	424 (13%)	162 (38%)	451 (12%)	206 (46%)
Once or twice per year	4930 (43%)	2212 (45%)	1414 (23%)	549 (39%)	418 (20%)	81 (19%)	1404 (41%)	819 (58%)	413 (12%)	180 (44%)	382 (10%)	159 (42%)
Once or twice per month	2549 (22%)	1094 (43%)	1828 (30%)	632 (35%)	261 (13%)	58 (22%)	1204 (35%)	660 (55%)	1003 (30%)	315 (31%)	790 (20%)	370 (47%)
Once per week or more	852 (8%)	407 (48%)	1820 (30%)	674 (37%)	411 (20%)	99 (24%)	374 (11%)	201 (54%)	1522 (45%)	583 (38%)	2277 (58%)	950 (42%)
Visiting people ^f^												
Never	4003 (35%)	1933 (48%)	978 (16%)	481 (49%)	1061 (52%)	227 (21%)	1035 (30%)	677 (65%)	575 (17%)	234 (41%)	662 (17%)	326 (49%)
Once or twice per year	4951 (44%)	2095 (42%)	1728 (28%)	683 (40%)	469 (23%)	85 (18%)	1508 (44%)	847 (56%)	608 (18%)	219 (36%)	534 (14%)	283 (53%)
Once or twice per month	1635 (14%)	704 (43%)	1706 (28%)	584 (34%)	266 (13%)	56 (21%)	736 (21%)	393 (53%)	1109 (33%)	393 (35%)	963 (25%)	449 (47%)
Once per week or more	766 (7%)	353 (46%)	1759 (29%)	577 (33%)	256 (13%)	64 (25%)	153 (5%)	72 (47%)	1061 (32%)	390 (37%)	1741 (45%)	627 (36%)
Socializing with coworkers												
Never	3182 (28%)	1526 (48%)	2540 (41%)	957 (38%)	1504 (73%)	333 (22%)	1658 (48%)	1093 (66%)	^d^		1604 (41%)	844 (53%)
Once or twice per year	2860 (25%)	1271 (44%)	1416 (23%)	567 (40%)	280 (14%)	41 (15%)	806 (23%)	425 (53%)			389 (10%)	167 (43%)
Once or twice per month	2530 (22%)	1080 (43%)	1102 (18%)	394 (36%)	142 (7%)	29 (20%)	562 (16%)	276 (49%)			602 (15%)	184 (31%)
Once per week or more	2778 (25%)	1206 (43%)	1113 (18%)	407 (37%)	126 (6%)	29 (23%)	420 (12%)	203 (48%)			1305 (34%)	490 (38%)
Social activities outside of home												
Never	2130 (19%)	1018 (48%)	1319 (21%)	528 (40%)	960 (47%)	192 (20%)	1244 (36%)	744 (60%)	789 (24%)	274 (35%)	505 (13%)	225 (45%)
Once or twice per year	6767 (60%)	2881 (43%)	3424 (56%)	1327 (39%)	630 (31%)	155 (25%)	1003 (29%)	597 (60%)	753 (22%)	265 (35%)	668 (17%)	283 (42%)
Once or twice per month	2002 (18%)	928 (46%)	1133 (18%)	374 (33%)	292 (14%)	56 (19%)	710 (21%)	385 (54%)	1024 (31%)	375 (37%)	994 (26%)	550 (55%)
Once per week or more	456 (4%)	258 (57%)	294 (5%)	95 (32%)	170 (8%)	29 (17%)	483 (14%)	266 (55%)	796 (24%)	326 (41%)	1733 (44%)	627 (36%)
Marital status												
Coupled	9488 (84%)	4123 (44%)	4631 (75%)	1651 (36%)	1252 (61%)	244 (20%)	1954 (57%)	1035 (53%)	1784 (53%)	599 (34%)	2224 (57%)	879 (40%)
Uncoupled	1867 (16%)	962 (52%)	1540 (25%)	674 (44%)	800 (39%)	188 (24%)	1492 (43%)	962 (65%)	1578 (47%)	641 (41%)	1676 (43%)	806 (48%)
**Environmental Factors**												
Location												
Urban	5347 (47%)	2859 (54%)	1535 (25%)	597 (39%)	1478 (72%)	348 (24%)	2608 (76%)	1537 (59%)	2247 (67%)	820 (37%)	1568 (40%)	735 (47%)
Rural	6008 (53%)	2226 (37%)	4636 (75%)	1728 (37%)	574 (28%)	84 (15%)	838 (24%)	460 (55%)	1114 (33%)	419 (38%)	2332 (60%)	950 (41%)
Personal motorized transport												
No	5165 (46%)	2189 (42%)	4564 (74%)	1773 (39%)	1308 (64%)	278 (21%)	1966 (57%)	1233 (63%)	2475 (74%)	980 (40%)	3685 (95%)	1596 (43%)
Yes	6190 (55%)	2896 (47%)	1607 (26%)	552 (34%)	743 (36%)	153 (21%)	1480 (43%)	764 (52%)	887 (26%)	260 (29%)	215 (6%)	89 (41%)
Computer in household												
No	8827 (78%)	3736 (42%)	5873 (95%)	2213 (38%)	1737 (85%)	354 (20%)	2782 (81%)	1649 (59%)	2894 (86%)	1100 (38%)	3714 (96%)	1603 (43%)
Yes	2528 (22%)	1349 (53%)	298 (5%)	112 (38%)	314 (15%)	77 (25%)	664 (19%)	348 (52%)	468 (14%)	140 (30%)	177 (5%)	76 (43%)
Safety out on the street after dark												
Completely/very safe	6806 (60%)	2992 (44%)	3763 (61%)	1583 (42%)	724 (35%)	154 (21%)	537 (16%)	291 (54%)	508 (15%)	233 (46%)	2851 (73%)	1134 (40%)
Moderately safe	2947 (26%)	1343 (46%)	1467 (24%)	463 (32%)	501 (24%)	101 (20%)	1005 (30%)	555 (55%)	648 (19%)	268 (41%)	719 (18%)	384 (53%)
Slightly safe/not at all	1591 (14%)	746 (47%)	941 (15%)	279 (30%)	827 (40%)	177 (21%)	1865 (55%)	1125 (60%)	2206 (66%)	739 (34%)	330 (9%)	167 (51%)
Safety when home alone												
Completely/very safe	9527 (84%)	4250 (45%)	4147 (67%)	1687 (41%)	1139 (56%)	255 (22%)	1494 (43%)	825 (55%)	801 (24%)	345 (43%)	3306 (85%)	1413 (43%)
Moderately safe	1482 (13%)	681 (46%)	1457 (24%)	455 (31%)	424 (21%)	87 (21%)	1132 (33%)	645 (57%)	917 (27%)	338 (37%)	425 (11%)	192 (45%)
Slightly safe/not at all	339 (3%)	151 (45%)	567 (9%)	183 (32%)	489 (24%)	90 (18%)	820 (24%)	527 (64%)	1642 (49%)	556 (34%)	169 (4%)	80 (47%)

^a^ Distribution of the factors in each country’s sample. ^b^ Percentages of people sedentary of ≥4 h/day for each factor level. ^c^ Education levels for some countries collapsed (e.g., secondary/high and college or more for India) due to low frequencies. ^d^ Factor omitted from the analyses due to large amounts of missing or implausible data. ^e^ Range: 0–10 for words recalled immediately (higher scores = higher function) and words lost with delay (higher scores = lower function), 0–5 for IADL and ADL (higher scores = lower function), and 0–100 for disability (higher scores = lower function) and quality of life (higher scores = higher function). ^f^ Visiting people in different neighborhoods or them coming to visit.

**Table 4:** Multivariate associations of ≥4 h/day sedentary behavior with demographic, health and health risk, functional, interpersonal, and environmental factors.

Factors	China		India		Mexico		Russian Federation		South Africa		Ghana	
	OR (95% CI) ^a^	*p* ^a^	OR (95% CI) ^a^	*p* ^a^	OR (95% CI) ^a^	*p* ^a^	OR (95% CI) ^a^	*p* ^a^	OR (95% CI) ^a^	*p* ^a^	OR (95% CI) ^a^	*p* ^a^
**Demographic Factors**												
Age (Ref: 50–59)												
60–69	1.22 (1.10, 1.35)	<0.0001	1.05 (0.92, 1.20)	0.0070	1.20 (0.85, 1.71)	0.0139	1.24 (1.02, 1.52)	<0.0001	1.12 (0.93, 1.36)	0.0802	1.00 (0.83, 1.19)	0.0109
70–79	1.56 (1.37, 1.77)		1.29 (1.09, 1.54)		1.43 (0.98, 2.10)		1.25 (0.99, 1.59)		1.35 (1.07, 1.71)		1.25 (1.02, 1.54)	
80+	2.16 (1.73, 2.71)		1.44 (1.08, 1.92)		2.07 (1.30, 3.28)		2.58 (1.80, 3.75)		1.26 (0.90, 1.76)		1.52 (1.13, 2.06)	
Sex (Ref: Male)												
Female	1.03 (0.94, 1.13)	0.5679	0.72 (0.62, 0.83)	<0.0001	0.71 (0.54, 0.93)	0.0131	0.94 (0.79, 1.11)	0.4550	0.92 (0.77, 1.08)	0.2961	1.12 (0.94, 1.35)	0.2135
Education completed (Ref: Lowest level, country-specific) ^b^												
Less than primary school	1.02 (0.90, 1.16)	0.0042	^c^		1.15 (0.85, 1.58)	0.0415			^d^			
Primary school	1.06 (0.93, 1.21)				0.98 (0.67, 1.43)						1.06 (0.84, 1.35)	0.7595
Secondary/high school	1.18 (1.03, 1.35)				1.82 (1.16, 2.85)		0.79 (0.59, 1.03)	0.0010			0.96 (0.79, 1.17)	
College or more	1.50 (1.20, 1.90)				1.41 (0.88, 2.24)		1.09 (0.79, 1.50)					
Employment (Ref: Working)												
Not working	1.51 (1.35, 1.69)	<0.0001	1.97 (1.70, 2.28)	<0.0001	0.99 (0.73, 1.35)	0.0759	1.12 (0.87, 1.44)	0.6011	1.66 (1.35, 2.04)	<0.0001	1.67 (1.36, 2.04)	<0.0001
Retired/too old to work	1.30 (1.16, 1.46)		2.20 (1.83, 2.64)		1.52 (0.98, 2.34)		1.09 (0.89, 1.35)		1.72 (1.37, 2.17)		2.05 (1.60, 2.65)	
Household wealth (Ref: 1st (high) quintile)												
2nd	0.95 (0.83, 1.09)	<0.0001	0.66 (0.55, 0.79)	0.0001	^c^		^c^		0.77 (0.59, 1.02)	0.0122	0.98 (0.78, 1.22)	0.2742
3rd	0.70 (0.59, 0.82)		0.71 (0.58, 0.88)						0.82 (0.60, 1.11)		0.82 (0.64, 1.04)	
4th	0.60 (0.50, 0.71)		0.76 (0.61, 0.93)						1.11 (0.81, 1.52)		0.92 (0.71, 1.18)	
5th (low) quintile	0.47 (0.39, 0.57)		0.66 (0.53, 0.83)						0.83 (0.60, 1.15)		1.03 (0.79, 1.34)	
**Health and Health-Risk Factors**												
Body mass index (Ref: Normal weight)												
Underweight	1.12 (0.93, 1.36)	<0.0001	1.03 (0.90, 1.16)	0.8483	^d^		^d^		^d^		1.04 (0.85, 1.28)	0.0445
Overweight	1.24 (1.13, 1.35)		0.94 (0.78, 1.13)								1.21 (0.99, 1.46)	
Obese	1.36 (1.14, 1.63)		0.97 (0.69, 1.35)								1.40 (1.08, 1.82)	
Alcohol use (Ref: Never drunk)												
Drunk in the past	0.99 (0.87, 1.13)	0.3854	1.12 (0.92, 1.36)	<0.0001	^c^		1.50 (1.25, 1.79)	<0.0001	^c^		1.29 (1.09, 1.53)	0.0164
≤1 drink per day	0.87 (0.74, 1.02)		0.45 (0.33, 0.60)				1.39 (1.11, 1.75)				1.01 (0.81, 1.26)	
>1 drink per day	1.00 (0.87, 1.15)		1.13 (0.71, 1.78)				1.98 (1.37, 2.88)				1.04 (0.81, 1.32)	
Smoking and tobacco use (Ref: No)												
Less than daily	^c^		0.86 (0.61, 1.19)	0.0336	^c^		^c^		^c^		^c^	
Daily			1.15 (1.02, 1.30)									
Non-communicable diseases (Ref: 0 diseases)												
1 disease	1.13 (1.02, 1.24)	0.0036	1.13 (0.99, 1.29)	0.0361	^c^		1.00 (0.81, 1.23)	0.0999	^c^		0.95 (0.80, 1.13)	0.2315
2 diseases	1.24 (1.10, 1.41)		0.95 (0.79, 1.13)				1.10 (0.89, 1.37)				1.09 (0.82, 1.44)	
3+ diseases	1.08 (0.91, 1.27)		0.84 (0.66, 1.05)				1.29 (1.02, 1.63)				1.61 (0.97, 2.72)	
Pain (Ref: None)												
Mild	0.98 (0.90, 1.08)	0.8042	^c^		0.91 (0.68, 1.22)	0.7689	1.17 (0.98, 1.41)	0.2060	^c^		^c^	
Moderate	1.05 (0.92, 1.21)				1.02 (0.74, 1.40)		1.09 (0.88, 1.35)					
Severe/extreme	1.02 (0.79, 1.33)				1.14 (0.74, 1.75)		0.93 (0.70, 1.24)					
Self-rated health (Ref: Good/very good)												
Moderate	1.02 (0.93, 1.13)	<0.0001	0.74 (0.65, 0.85)	<0.0001	^c^		1.15 (0.91, 1.45)	<0.0001	0.75 (0.63, 0.91)	0.0102	1.43 (1.21, 1.68)	<0.0001
Bad/very bad	1.45 (1.26, 1.67)		1.03 (0.85, 1.25)				2.62 (1.93, 3.55)		0.79 (0.60, 1.05)		2.18 (1.71, 2.79)	
**Functional Factors**												
Mobility (Ref: Mobility)												
Dismobility	1.24 (1.02, 1.50)	0.0306	1.33 (1.13, 1.58)	0.0008	1.34 (1.00, 1.78)	0.0473	^d^		^d^		1.15 (0.98, 1.35)	0.0884
Distance vision impairment (Ref: Mild or none)												
Moderate or greater	^c^		^c^		^d^		^d^		1.36 (1.08, 1.70)	0.0079	1.27 (1.02, 1.59)	0.0344
Near vision impairment (Ref: Mild or none)												
Moderate or greater	1.24 (1.13, 1.35)	<0.0001	^c^		^d^		^d^		^c^		1.14 (0.97, 1.35)	0.1035
Verbal learning and memory												
Words recalled immediately ^e^	1.08 (1.05, 1.11)	<0.0001	^c^		^c^		^c^		0.89 (0.85, 0.94)	<0.0001	0.95 (0.90, 1.01)	0.0929
Words lost with delay ^e^	^c^		^c^		0.90 (0.85, 0.96)	0.0007	^c^		^d^		^c^	
IADL ^e^	1.45 (1.30, 1.62)	<0.0001	1.08 (1.00, 1.18)	0.0589	1.65 (1.42, 1.92)	<0.0001	^c^		1.18 (1.06, 1.32)	0.0034	0.98 (0.87, 1.11)	0.7491
Quality of Life ^e^	1.00 (1.00, 1.01)	0.4089	0.98 (0.97, 0.98)	<0.0001	^c^		^c^		0.98 (0.97, 0.98)	<0.0001	0.99 (0.99, 1.00)	0.0129
**Interpersonal Factors**												
Emotional loneliness (Ref: No)												
Yes	^c^		^c^		1.36 (1.02, 1.81)	0.0339	1.37 (1.08, 1.75)	0.0111	1.01 (0.79, 1.30)	0.9179	2.60 (2.03, 3.34)	<0.0001
Friends visiting home (Ref: Never)												
Once or twice per year	1.19 (1.07, 1.33)	0.0009	1.06 (0.88, 1.28)	0.0977	^c^		^c^		1.36 (1.01, 1.84)	0.0018	0.87 (0.62, 1.22)	0.0011
Once or twice per month	1.18 (1.04, 1.34)		1.01 (0.84, 1.22)						0.83 (0.64, 1.07)		1.24 (0.92, 1.67)	
Once per week or more	1.38 (1.16, 1.65)		1.22 (1.01, 1.48)						0.92 (0.72, 1.19)		1.47 (1.11, 1.95)	
Visiting people (Ref: Never) ^f^												
Once or twice per year	0.87 (0.79, 0.96)	0.0034	0.78 (0.65, 0.94)	<0.0001	^c^		^c^		^c^		1.14 (0.85, 1.52)	0.0088
Once or twice per month	0.84 (0.74, 0.97)		0.60 (0.49, 0.73)								0.89 (0.68, 1.17)	
Once per week or more	1.07 (0.90, 1.27)		0.51 (0.42, 0.62)								0.74 (0.57, 0.97)	
Socializing with coworkers (Ref: Never)												
Once or twice per year	^c^		1.34 (1.15, 1.56)	0.0003	0.63 (0.43, 0.91)	0.0343	0.80 (0.66, 0.97)	0.0437	^d^		0.91 (0.71, 1.18)	<0.0001
Once or twice per month			1.27 (1.07, 1.50)		1.08 (0.68, 1.68)		0.78 (0.63, 0.98)				0.48 (0.38, 0.60)	
Once per week or more			1.32 (1.12, 1.57)		1.37 (0.85, 2.17)		0.78 (0.61, 1.00)				0.78 (0.65, 0.94)	
Social activities outside of home (Ref: Never)												
Once or twice per year	0.96 (0.87, 1.08)	0.0005	^c^		^c^		^c^		1.20 (0.95, 1.51)	<0.0001	1.29 (0.98, 1.70)	<0.0001
Once or twice per month	1.04 (0.91, 1.19)								1.56 (1.26, 1.94)		2.43 (1.86, 3.19)	
Once per week or more	1.48 (1.19, 1.84)								1.61 (1.27, 2.04)		1.37 (1.06, 1.77)	
Marital status (Ref: Coupled)												
Uncoupled	1.18 (1.05, 1.32)	0.0043	1.13 (0.99, 1.30)	0.0759	1.04 (0.80, 1.34)	0.7628	1.17 (0.99, 1.38)	0.0708	1.09 (0.92, 1.28)	0.3396	0.87 (0.72, 1.04)	0.1171
**Environmental Factors**												
Location (Ref: Urban)												
Rural	0.73 (0.65, 0.81)	<0.0001	^c^		0.56 (0.41, 0.74)	<0.0001	^c^		^c^		0.89 (0.75, 1.05)	0.1615
Personal motorized transport (Ref: No)												
Yes	0.88 (0.79, 0.97)	0.0112	0.80 (0.67, 0.94)	0.0071	^c^		0.85 (0.73, 0.99)	0.0394	0.80 (0.63, 1.01)	0.059	^c^	
Computer in household (Ref: No)												
Yes	1.00 (0.88, 1.15)	0.9579	^c^		^c^		1.07 (0.88, 1.30)	0.4848	1.00 (0.77, 1.30)	0.986	^c^	
Safety out on the street after dark (Ref: Completely/very safe)												
Moderately safe	^c^		0.58 (0.50, 0.66)	<0.0001	^c^		^c^		0.76 (0.59, 0.97)	<0.0001	1.39 (1.15, 1.68)	0.0010
Slightly safe/not at all			0.50 (0.42, 0.59)						0.55 (0.44, 0.68)		1.31 (1.01, 1.69)	
Safety when home alone (Ref: Completely/very safe)												
Moderately safe	^c^		^c^		^c^		1.03 (0.87, 1.22)	0.1886	^c^		^c^	
Slightly safe/not at all							1.19 (0.98, 1.44)					

^a^ Odds ratios (95% confidence intervals) and p values for those factors that were selected in the fully adjusted final model for each country. ^b^ Education levels for some countries collapsed (e.g., secondary/high and college or more for India) due to low numbers. ^c^ Factor was not selected for the final model. ^d^ Factor omitted from the analyses due to large amounts of missing or implausible data. ^e^ Higher scores = higher function for words recalled immediately and quality of life; higher scores = lower function for words lost with delay and IADL. ^f^ Visiting people in different neighborhoods or them coming to visit.

## Supplementary Materials

The following tables are available online at http://www.mdpi.com/1660-4601/15/5/908/s1, Table S1: Univariate associations of meeting physical activity guidelines with demographic, health and health risk, functional, interpersonal, and environmental factors, Table S2: Univariate associations of ≥4 h/day sedentary behavior with demographic, health and health risk, functional, interpersonal, and environmental factors.
