# Supplementary material for: Factors Associated with Physical Activity and Sedentary Behavior in Older Adults from Six Low- and Middle-Income Countries
Source: Int J Environ Res Public Health. 2018 May 3;15(5):908. doi: 10.3390/ijerph15050908 (PMC5981947; doi:10.3390/ijerph15050908)
Supplement: Supplementary file 1 [file ijerph-15-00908-s001.pdf]

**Table S1.** Univariate associations of meeting physical activity guidelines with demographic, health and health risk, functional, interpersonal, and environmental factors.

| Factors | China | | India | | Mexico | | Russian Federation | | South Africa | | Ghana | |
|---|---|---|---|---|---|---|---|---|---|---|---|---|
| | OR (95% CI) [a] | p [a] | OR (95% CI) [a] | p [a] | OR (95% CI) [a] | p [a] | OR (95% CI) [a] | p [a] | OR (95% CI) [a] | p [a] | OR (95% CI) [a] | p [a] |
| **Demographic Factors** | | | | | | | | | | | | |
| Age (Ref: 50-59) | | | | | | | | | | | | |
| 60-69 | 0.72 (0.66, 0.79) | <0.0001 | 0.57 (0.50, 0.64) | <0.0001 | 0.82 (0.64, 1.06) | <0.0001 | 0.63 (0.53, 0.75) | <0.0001 | 0.47 (0.39, 0.57) | <0.0001 | 0.73 (0.62, 0.86) | <0.0001 |
| 70-79 | 0.41 (0.37, 0.46) | | 0.28 (0.24, 0.33) | | 0.45 (0.34, 0.60) | | 0.34 (0.29, 0.41) | | 0.31 (0.23, 0.41) | | 0.43 (0.36, 0.51) | |
| 80+ | 0.18 (0.14, 0.24) | | 0.12 (0.08, 0.18) | | 0.17 (0.10, 0.27) | | 0.14 (0.11, 0.19) | | 0.24 (0.14, 0.37) | | 0.28 (0.22, 0.36) | |
| Sex (Ref: Male) | | | | | | | | | | | | |
| Female | 0.74 (0.68, 0.80) | <0.0001 | 0.86 (0.77, 0.95) | 0.004 | 0.63 (0.52, 0.77) | <0.0001 | 0.84 (0.73, 0.97) | 0.0173 | 0.66 (0.56, 0.79) | <0.0001 | 0.62 (0.54, 0.71) | <0.0001 |
| Education completed (Ref: Lowest level, country-specific) [b] | | | | | | | | | | | | |
| Less than primary school | 1.36 (1.20, 1.53) | <0.0001 | 1.10 (0.93, 1.31) | 0.009 | 1.15 (0.88, 1.51) | 0.0576 | | | [c] | | | |
| Primary school | 1.29 (1.14, 1.45) | | 1.28 (1.10, 1.50) | | 1.25 (0.92, 1.70) | | | | | | 0.94 (0.76, 1.16) | 0.0024 |
| Secondary/high school | 0.83 (0.75, 0.93) | | 1.15 (1.01, 1.31) | | 1.48 (1.01, 2.16) | | 2.56 (2.01, 3.26) | <0.0001 | | | 0.76 (0.65, 0.89) | |
| College or more | 0.28 (0.20, 0.37) | | | | 0.77 (0.49, 1.18) | | 2.83 (2.16, 3.73) | | | | | |
| Employment (Ref: Working) | | | | | | | | | | | | |
| Not working | 0.37 (0.33, 0.41) | <0.0001 | 0.36 (0.32, 0.40) | <0.0001 | 0.28 (0.23, 0.35) | <0.0001 | 0.46 (0.37, 0.56) | <0.0001 | 0.31 (0.26, 0.38) | <0.0001 | 0.27 (0.23, 0.32) | <0.0001 |
| Retired/too old to work | 0.27 (0.24, 0.29) | | 0.17 (0.14, 0.21) | | 0.14 (0.09, 0.22) | | 0.40 (0.35, 0.47) | | 0.23 (0.19, 0.29) | | 0.14 (0.11, 0.18) | |
| Household wealth (Ref: 1st (high) quintile) | | | | | | | | | | | | |
| 2nd | 1.69 (1.47, 1.94) | <0.0001 | 1.12 (0.95, 1.31) | <0.0001 | 0.97 (0.71, 1.32) | 0.5732 | 1.02 (0.83, 1.26) | <0.0001 | 0.90 (0.69, 1.19) | 0.0015 | 1.52 (1.24, 1.86) | <0.0001 |
| 3rd | 1.93 (1.69, 2.22) | | 1.41 (1.20, 1.66) | | 0.95 (0.69, 1.31) | | 0.71 (0.58, 0.87) | | 1.04 (0.79, 1.36) | | 2.38 (1.93, 2.94) | |
| 4th | 2.48 (2.17, 2.84) | | 1.69 (1.44, 1.99) | | 0.81 (0.59, 1.10) | | 0.55 (0.44, 0.67) | | 1.28 (0.99, 1.67) | | 2.04 (1.66, 2.51) | |
| 5th (low) quintile | 2.13 (1.86, 2.44) | | 1.78 (1.51, 2.11) | | 1.03 (0.76, 1.39) | | 0.44 (0.35, 0.54) | | 1.48 (1.14, 1.92) | | 2.26 (1.83, 2.78) | |
| **Health and Health-Risk Factors** | | | | | | | | | | | | |
| Body mass index (Ref: Normal weight) | | | | | | | | | | | | |
| Underweight | 0.81 (0.66, 0.98) | <0.0001 | 0.91 (0.82, 1.03) | 0.0106 | [c] | | [c] | | [c] | | 0.73 (0.61, 0.89) | <0.0001 |
| Overweight | 0.76 (0.69, 0.83) | | 0.87 (0.73, 1.03) | | | | | | | | 0.61 (0.52, 0.73) | |
| Obese | 0.60 (0.49, 0.73) | | 0.61 (0.43, 0.84) | | | | | | | | 0.49 (0.39, 0.62) | |
| Alcohol use (Ref: Never drunk) | | | | | | | | | | | | |
| Drunk in the past | 1.53 (1.35, 1.73) | <0.0001 | 1.10 (0.92, 1.32) | <0.0001 | 1.40 (1.13, 1.72) | 0.0005 | 1.53 (1.30, 1.80) | <0.0001 | 1.24 (0.97, 1.56) | 0.2298 | 1.06 (0.91, 1.24) | <0.0001 |
| ≤ 1 drink per day | 1.53 (1.32, 1.78) | | 1.48 (1.16, 1.87) | | 1.33 (0.93, 1.88) | | 2.39 (1.94, 2.94) | | 0.87 (0.62, 1.20) | | 1.31 (1.08, 1.60) | |
| > 1 drink per day | 2.33 (2.06, 2.62) | | 2.03 (1.33, 3.14) | | 2.68 (1.42, 5.00) | | 2.53 (1.80, 3.58) | | 0.98 (0.66, 1.41) | | 2.06 (1.65, 2.59) | |
| Smoking and tobacco use (Ref: No) | | | | | | | | | | | | |
| Less than daily | 1.28 (0.99, 1.64) | <0.0001 | 0.68 (0.48, 0.96) | <0.0001 | 1.11 (0.73, 1.66) | 0.7301 | 0.67 (0.37, 1.19) | <0.0001 | 0.42 (0.23, 0.71) | 0.0008 | 1.83 (1.21, 2.85) | <0.0001 |
| Daily | 1.67 (1.52, 1.82) | | 1.44 (1.30, 1.61) | | 1.10 (0.82, 1.47) | | 1.51 (1.25, 1.81) | | 0.77 (0.62, 0.95) | | 1.54 (1.23, 1.94) | |
| Non-communicable diseases (Ref: 0 diseases) | | | | | | | | | | | | |
| 1 disease | 0.71 (0.65, 0.78) | <0.0001 | 0.77 (0.69, 0.87) | <0.0001 | 0.73 (0.58, 0.92) | <0.0001 | 1.08 (0.89, 1.31) | <0.0001 | 0.70 (0.58, 0.85) | <0.0001 | 0.67 (0.57, 0.78) | <0.0001 |
| 2 diseases | 0.47 (0.41, 0.53) | | 0.57 (0.48, 0.67) | | 0.66 (0.49, 0.87) | | 0.81 (0.67, 0.99) | | 0.41 (0.30, 0.55) | | 0.41 (0.32, 0.53) | |
| 3+ diseases | 0.34 (0.28, 0.41) | | 0.39 (0.31, 0.50) | | 0.39 (0.25, 0.60) | | 0.53 (0.44, 0.64) | | 0.30 (0.19, 0.45) | | 0.40 (0.26, 0.63) | |

**Table S1.** *Cont.*

| Factors | China | | India | | Mexico | | Russian Federation | | South Africa | | Ghana | |
|---|---|---|---|---|---|---|---|---|---|---|---|---|
| | OR (95% CI) [a] | p [a] | OR (95% CI) [a] | p [a] | OR (95% CI) [a] | p [a] | OR (95% CI) [a] | p [a] | OR (95% CI) [a] | p [a] | OR (95% CI) [a] | p [a] |
| **Health and Health-Risk Factors (*Cont.*)** | | | | | | | | | | | | |
| Pain (Ref: None) | | | | | | | | | | | | |
| Mild | 1.14 (1.05, 1.25) | <0.0001 | 0.84 (0.74, 0.96) | <0.0001 | 0.95 (0.75, 1.19) | <0.0001 | 0.82 (0.69, 0.97) | <0.0001 | 0.58 (0.48, 0.71) | <0.0001 | 1.90 (1.58, 2.29) | <0.0001 |
| Moderate | 1.16 (1.03, 1.32) | | 0.78 (0.67, 0.91) | | 0.54 (0.40, 0.71) | | 0.69 (0.58, 0.83) | | 0.36 (0.28, 0.46) | | 1.20 (0.99, 1.45) | |
| Severe/extreme | 0.56 (0.42, 0.74) | | 0.70 (0.59, 0.82) | | 0.41 (0.26, 0.62) | | 0.48 (0.38, 0.59) | | 0.38 (0.27, 0.52) | | 0.82 (0.66, 1.02) | |
| Self-rated health (Ref: Good/very good) | | | | | | | | | | | | |
| Moderate | 0.77 (0.70, 0.84) | <0.0001 | 0.67 (0.60, 0.76) | <0.0001 | 0.69 (0.56, 0.85) | <0.0001 | 0.81 (0.66, 1.00) | <0.0001 | 0.40 (0.34, 0.49) | <0.0001 | 0.66 (0.57, 0.77) | <0.0001 |
| Bad/very bad | 0.74 (0.66, 0.82) | | 0.46 (0.40, 0.54) | | 0.54 (0.38, 0.75) | | 0.30 (0.24, 0.38) | | 0.27 (0.20, 0.36) | | 0.30 (0.24, 0.36) | |
| **Functional Factors** | | | | | | | | | | | | |
| Mobility (Ref: Mobility) | | | | | | | | | | | | |
| Dismobility | 0.30 (0.24, 0.38) | <0.0001 | 0.46 (0.38, 0.54) | <0.0001 | 0.35 (0.27, 0.46) | <0.0001 | [c] | | [c] | | 0.70 (0.61, 0.81) | <0.0001 |
| Distance vision impairment (Ref: Mild or none) | | | | | | | | | | | | |
| Moderate or greater | 0.60 (0.52, 0.69) | <0.0001 | 0.56 (0.48, 0.64) | <0.0001 | [c] | | [c] | | 0.55 (0.40, 0.74) | <0.0001 | 0.88 (0.72, 1.07) | 0.1893 |
| Near vision impairment (Ref: Mild or none) | | | | | | | | | | | | |
| Moderate or greater | 0.93 (0.86, 1.01) | 0.1010 | 0.86 (0.78, 0.96) | 0.0065 | [c] | | [c] | | 0.92 (0.77, 1.09) | 0.3381 | 0.79 (0.68, 0.91) | 0.0012 |
| Verbal learning and memory | | | | | | | | | | | | |
| Words recalled immediately [d] | 0.98 (0.96, 1.00) | 0.0870 | 1.13 (1.09, 1.17) | <0.0001 | 1.14 (1.06, 1.23) | 0.0002 | 1.34 (1.28, 1.40) | <0.0001 | 1.28 (1.21, 1.36) | <0.0001 | 1.27 (1.21, 1.34) | <0.0001 |
| Words lost with delay [d] | 1.01 (0.99, 1.04) | 0.4020 | 0.98 (0.94, 1.01) | 0.2101 | 1.03 (0.99, 1.09) | 0.1711 | 1.00 (0.95, 1.04) | 0.8524 | [c] | | 0.95 (0.91, 0.99) | 0.0072 |
| IADL [d] | 0.39 (0.34, 0.44) | <0.0001 | 0.65 (0.61, 0.69) | <0.0001 | 0.45 (0.37, 0.53) | <0.0001 | 0.43 (0.39, 0.47) | <0.0001 | 0.38 (0.31, 0.45) | <0.0001 | 0.58 (0.53, 0.63) | <0.0001 |
| ADL [d] | 0.39 (0.34, 0.45) | <0.0001 | 0.54 (0.49, 0.59) | <0.0001 | 0.41 (0.34, 0.48) | <0.0001 | 0.39 (0.35, 0.44) | <0.0001 | 0.40 (0.34, 0.47) | <0.0001 | 0.50 (0.45, 0.56) | <0.0001 |
| Disability [d] | 0.97 (0.97, 0.98) | <0.0001 | 0.98 (0.98, 0.98) | <0.0001 | 0.96 (0.96, 0.97) | <0.0001 | 0.96 (0.96, 0.97) | <0.0001 | 0.96 (0.96, 0.97) | <0.0001 | 0.98 (0.97, 0.98) | <0.0001 |
| Quality of Life [d] | 1.01 (1.00, 1.01) | <0.0001 | 1.01 (1.01, 1.02) | <0.0001 | 1.02 (1.02, 1.03) | <0.0001 | 1.02 (1.02, 1.03) | <0.0001 | 1.02 (1.01, 1.02) | <0.0001 | 1.02 (1.01, 1.02) | <0.0001 |
| **Interpersonal Factors** | | | | | | | | | | | | |
| Emotional loneliness (Ref: No) | | | | | | | | | | | | |
| Yes | 0.76 (0.64, 0.91) | 0.0040 | 0.84 (0.72, 0.97) | 0.0150 | 0.69 (0.52, 0.90) | 0.0064 | 0.55 (0.45, 0.68) | <0.0001 | 0.77 (0.56, 1.04) | 0.0957 | 0.57 (0.46, 0.70) | <0.0001 |
| Friends visiting home (Ref: Never) | | | | | | | | | | | | |
| Once or twice per year | 0.96 (0.87, 1.07) | <0.0001 | 1.03 (0.87, 1.21) | 0.0030 | 1.35 (1.04, 1.75) | <0.0001 | 1.88 (1.51, 2.33) | <0.0001 | 2.11 (1.46, 3.06) | 0.0001 | 1.38 (1.03, 1.83) | <0.0001 |
| Once or twice per month | 1.31 (1.17, 1.47) | | 1.21 (1.04, 1.42) | | 1.50 (1.10, 2.03) | | 1.86 (1.49, 2.33) | | 1.81 (1.32, 2.53) | | 1.84 (1.44, 2.35) | |
| Once per week or more | 1.75 (1.49, 2.05) | | 1.27 (1.08, 1.48) | | 1.79 (1.39, 2.31) | | 2.27 (1.72, 3.01) | | 1.97 (1.45, 2.72) | | 4.66 (3.75, 5.81) | |
| Visiting people (Ref: Never) [e] | | | | | | | | | | | | |
| Once or twice per year | 1.22 (1.12, 1.34) | <0.0001 | 1.22 (1.03, 1.44) | 0.0010 | 1.35 (1.06, 1.72) | 0.0479 | 1.65 (1.41, 1.94) | <0.0001 | 2.32 (1.72, 3.16) | <0.0001 | 1.57 (1.23, 1.99) | <0.0001 |
| Once or twice per month | 1.41 (1.25, 1.60) | | 1.21 (1.02, 1.43) | | 1.35 (1.00, 1.81) | | 1.86 (1.54, 2.26) | | 1.42 (1.07, 1.91) | | 2.54 (2.06, 3.13) | |
| Once per week or more | 1.87 (1.59, 2.20) | | 1.41 (1.20, 1.67) | | 1.18 (0.86, 1.61) | | 2.23 (1.58, 3.18) | | 2.26 (1.71, 3.01) | | 5.52 (4.54, 6.73) | |
| Socializing with coworkers (Ref: Never) | | | | | | | | | | | | |
| Once or twice per year | 1.00 (0.89, 1.12) | <0.0001 | 1.25 (1.09, 1.43) | <0.0001 | 1.66 (1.26, 2.18) | <0.0001 | 2.03 (1.71, 2.41) | <0.0001 | [c] | | 1.52 (1.21, 1.91) | <0.0001 |
| Once or twice per month | 1.18 (1.05, 1.33) | | 1.45 (1.25, 1.69) | | 1.57 (1.07, 2.26) | | 2.33 (1.92, 2.84) | | | | 2.90 (2.37, 3.56) | |
| Once per week or more | 1.97 (1.77, 2.20) | | 2.41 (2.08, 2.80) | | 2.97 (2.04, 4.31) | | 2.95 (2.36, 3.70) | | | | 4.35 (3.69, 5.14) | |

**Table S1.** *Cont.*

| Factors | China | | India | | Mexico | | Russian Federation | | South Africa | | Ghana | |
|---|---|---|---|---|---|---|---|---|---|---|---|---|
| | OR (95% CI) [a] | p [a] | OR (95% CI) [a] | p [a] | OR (95% CI) [a] | p [a] | OR (95% CI) [a] | p [a] | OR (95% CI) [a] | p [a] | OR (95% CI) [a] | p [a] |
| **Interpersonal Factors (Cont.)** | | | | | | | | | | | | |
| Social activities outside of home (Ref: Never) | | | | | | | | | | | | |
| Once or twice per year | 1.61 (1.44, 1.81) | <0.0001 | 1.56 (1.36, 1.79) | <0.0001 | 1.48 (1.18, 1.86) | 0.0053 | 1.39 (1.17, 1.64) | <0.0001 | 1.96 (1.52, 2.53) | <0.0001 | 1.69 (1.33, 2.14) | <0.0001 |
| Once or twice per month | 1.71 (1.49, 1.96) | | 1.79 (1.52, 2.12) | | 1.29 (0.95, 1.73) | | 1.89 (1.57, 2.28) | | 1.66 (1.30, 2.12) | | 2.23 (1.78, 2.79) | |
| Once per week or more | 1.99 (1.60, 2.47) | | 1.97 (1.51, 2.56) | | 1.38 (0.96, 1.98) | | 2.39 (1.93, 2.97) | | 1.14 (0.87, 1.50) | | 3.45 (2.80, 4.26) | |
| Marital status (Ref: Coupled) | | | | | | | | | | | | |
| Uncoupled | 0.72 (0.64, 0.80) | <0.0001 | 0.70 (0.61, 0.79) | <0.0001 | 0.58 (0.47, 0.72) | <0.0001 | 0.61 (0.53, 0.70) | <0.0001 | 0.73 (0.61, 0.86) | 0.0002 | 0.56 (0.49, 0.63) | <0.0001 |
| **Environmental Factors** | | | | | | | | | | | | |
| Location (Ref: Urban) | | | | | | | | | | | | |
| Rural | 2.56 (2.35, 2.78) | <0.0001 | 1.55 (1.37, 1.76) | <0.0001 | 1.23 (1.00, 1.53) | 0.0542 | 1.46 (1.25, 1.71) | <0.0001 | 1.18 (0.99, 1.41) | 0.0620 | 2.60 (2.27, 2.98) | <0.0001 |
| Personal motorized transport (Ref: No) | | | | | | | | | | | | |
| Yes | 0.81 (0.75, 0.88) | <0.0001 | 0.68 (0.60, 0.76) | <0.0001 | 0.94 (0.76, 1.15) | 0.5207 | 1.58 (1.38, 1.81) | <0.0001 | 0.88 (0.72, 1.06) | 0.1774 | 0.40 (0.29, 0.53) | <0.0001 |
| Computer in household (Ref: No) | | | | | | | | | | | | |
| Yes | 0.45 (0.40, 0.50) | <0.0001 | 0.69 (0.53, 0.88) | 0.0030 | 0.78 (0.58, 1.03) | 0.0840 | 1.34 (1.13, 1.58) | 0.0010 | 1.05 (0.82, 1.33) | 0.6802 | 0.35 (0.25, 0.48) | <0.0001 |
| Safety out on the street after dark (Ref: Completely/very safe) | | | | | | | | | | | | |
| Moderately safe | 0.60 (0.54, 0.66) | <0.0001 | 0.80 (0.70, 0.90) | 0.0010 | 0.96 (0.74, 1.24) | 0.8757 | 1.05 (0.85, 1.29) | 0.2850 | 0.85 (0.64, 1.13) | 0.0912 | 0.87 (0.74, 1.03) | <0.0001 |
| Slightly safe/not at all | 0.89 (0.79, 1.00) | | 0.84 (0.72, 0.97) | | 0.94 (0.75, 1.18) | | 0.93 (0.77, 1.13) | | 0.78 (0.62, 0.98) | | 0.45 (0.35, 0.57) | |
| Safety when home alone (Ref: Completely/very safe) | | | | | | | | | | | | |
| Moderately safe | 0.83 (0.74, 0.94) | 0.0020 | 0.83 (0.73, 0.94) | 0.0010 | 0.83 (0.64, 1.07) | 0.2890 | 0.83 (0.71, 0.97) | 0.0600 | 0.61 (0.49, 0.77) | <0.0001 | 0.48 (0.39, 0.60) | <0.0001 |
| Slightly safe/not at all | 1.24 (0.98, 1.55) | | 0.77 (0.63, 0.92) | | 0.88 (0.69, 1.12) | | 0.92 (0.78, 1.09) | | 0.62 (0.50, 0.75) | | 0.29 (0.21, 0.40) | |

[a] Odds ratio (95% confidence interval) and p value from a univariate model including each factor separately. [b] Education levels for some countries collapsed (e.g., secondary/high and college or more for India) due to low numbers. [c] Factor omitted from the analyses due to large amounts of missing or implausible data. [d] Higher scores = higher function for words recalled immediately and quality of life; higher scores = lower function for words lost with delay, IADL, ADL, and disability. [e] Visiting people in different neighborhoods or them coming to visit.

**Table S2.** Univariate associations of ≥4hrs/day sedentary behavior with demographic, health and health risk, functional, interpersonal, and environmental factors.

| Factors | China | | India | | Mexico | | Russian Federation | | South Africa | | Ghana | |
|---|---|---|---|---|---|---|---|---|---|---|---|---|
| | OR (95% CI) [a] | p [a] | OR (95% CI) [a] | p [a] | OR (95% CI) [a] | p [a] | OR (95% CI) [a] | p [a] | OR (95% CI) [a] | p [a] | OR (95% CI) [a] | p [a] |
| **Demographic Factors** | | | | | | | | | | | | |
| Age (Ref: 50-59) | | | | | | | | | | | | |
| 60-69 | 1.31 (1.20, 1.43) | <0.0001 | 1.33 (1.18, 1.50) | <0.0001 | 1.23 (0.89, 1.72) | <0.0001 | 1.61 (1.36, 1.91) | <0.0001 | 1.31 (1.11, 1.54) | <0.0001 | 1.24 (1.06, 1.45) | <0.0001 |
| 70-79 | 1.87 (1.69, 2.07) | | 2.03 (1.75, 2.36) | | 1.79 (1.28, 2.53) | | 2.11 (1.77, 2.53) | | 1.73 (1.43, 2.11) | | 1.92 (1.62, 2.27) | |
| 80+ | 2.93 (2.45, 3.53) | | 2.85 (2.23, 3.66) | | 3.66 (2.51, 5.38) | | 5.39 (4.00, 7.38) | | 1.86 (1.39, 2.50) | | 2.84 (2.25, 3.59) | |
| Sex (Ref: Male) | | | | | | | | | | | | |
| Female | 1.19 (1.10, 1.28) | <0.0001 | 0.94 (0.85, 1.04) | 0.2254 | 0.83 (0.67, 1.03) | 0.0957 | 1.13 (0.98, 1.30) | 0.1005 | 1.10 (0.96, 1.27) | 0.1843 | 1.47 (1.30, 1.67) | <0.0001 |
| Education completed (Ref: Lowest level, country-specific) [b] | | | | | | | | | | | | |
| Less than primary school | 0.88 (0.78, 0.98) | <0.0001 | 1.06 (0.89, 1.25) | 0.1799 | 0.98 (0.74, 1.31) | 0.0865 | | | [c] | | | |
| Primary school | 0.98 (0.88, 1.09) | | 1.18 (1.01, 1.38) | | 0.79 (0.56, 1.12) | | | | | | 0.93 (0.76, 1.15) | 0.0048 |
| Secondary/high school | 1.22 (1.10, 1.35) | | 1.01 (0.89, 1.15) | | 1.37 (0.91, 2.04) | | 0.45 (0.35, 0.58) | <0.0001 | | | 0.78 (0.67, 0.90) | |
| College or more | 2.12 (1.74, 2.59) | | | | 1.20 (0.78, 1.83) | | 0.48 (0.36, 0.63) | | | | | |
| Employment (Ref: Working) | | | | | | | | | | | | |
| Not working | 1.81 (1.64, 2.00) | <0.0001 | 1.92 (1.72, 2.16) | <0.0001 | 1.29 (1.00, 1.67) | <0.0001 | 2.02 (1.63, 2.51) | <0.0001 | 2.03 (1.69, 2.45) | <0.0001 | 2.64 (2.22, 3.13) | <0.0001 |
| Retired/too old to work | 2.36 (2.17, 2.57) | | 2.55 (2.17, 3.00) | | 2.42 (1.65, 3.54) | | 2.13 (1.83, 2.48) | | 2.13 (1.76, 2.59) | | 3.61 (2.93, 4.46) | |
| Household wealth (Ref: 1st (high) quintile) | | | | | | | | | | | | |
| 2nd | 0.89 (0.80, 1.00) | <0.0001 | 0.78 (0.67, 0.91) | 0.0006 | 1.25 (0.89, 1.75) | 0.4631 | 1.07 (0.87, 1.32) | <0.0001 | 1.11 (0.89, 1.38) | <0.0001 | 0.93 (0.76, 1.13) | 0.0753 |
| 3rd | 0.69 (0.62, 0.78) | | 0.95 (0.81, 1.12) | | 1.13 (0.80, 1.61) | | 1.20 (0.97, 1.47) | | 1.26 (1.01, 1.58) | | 0.77 (0.63, 0.94) | |
| 4th | 0.56 (0.50, 0.63) | | 1.09 (0.93, 1.27) | | 1.07 (0.76, 1.50) | | 1.47 (1.19, 1.81) | | 1.76 (1.41, 2.20) | | 0.93 (0.76, 1.13) | |
| 5th (low) quintile | 0.51 (0.45, 0.58) | | 1.05 (0.89, 1.23) | | 0.93 (0.66, 1.32) | | 1.61 (1.30, 2.00) | | 1.49 (1.19, 1.87) | | 0.98 (0.81, 1.20) | |
| **Health and Health-Risk Factors** | | | | | | | | | | | | |
| Body mass index (Ref: Normal weight) | | | | | | | | | | | | |
| Underweight | 1.13 (0.94, 1.35) | <0.0001 | 1.22 (1.09, 1.36) | 0.0010 | [c] | | [c] | | [c] | | 1.25 (1.04, 1.50) | 0.0007 |
| Overweight | 1.36 (1.25, 1.48) | | 0.92 (0.77, 1.09) | | | | | | | | 1.22 (1.03, 1.44) | |
| Obese | 1.61 (1.37, 1.91) | | 0.95 (0.69, 1.30) | | | | | | | | 1.48 (1.19, 1.85) | |
| Alcohol use (Ref: Never drunk) | | | | | | | | | | | | |
| Drunk in the past | 0.96 (0.85, 1.08) | <0.0001 | 1.34 (1.12, 1.58) | <0.0001 | 1.23 (0.98, 1.54) | 0.1163 | 1.34 (1.14, 1.57) | 0.0002 | 1.01 (0.82, 1.23) | 0.0383 | 1.27 (1.10, 1.48) | <0.0001 |
| ≤ 1 drink per day | 0.70 (0.60, 0.80) | | 0.47 (0.36, 0.61) | | 0.95 (0.63, 1.39) | | 0.95 (0.78, 1.17) | | 1.00 (0.76, 1.30) | | 0.85 (0.70, 1.03) | |
| > 1 drink per day | 0.73 (0.65, 0.82) | | 1.03 (0.67, 1.57) | | 0.54 (0.18, 1.27) | | 1.23 (0.88, 1.74) | | 1.55 (1.15, 2.08) | | 0.75 (0.61, 0.92) | |
| Smoking and tobacco use (Ref: No) | | | | | | | | | | | | |
| Less than daily | 0.68 (0.53, 0.86) | <0.0001 | 0.91 (0.67, 1.24) | 0.2087 | 1.08 (0.68, 1.66) | 0.6034 | 0.98 (0.55, 1.78) | 0.0746 | 0.78 (0.53, 1.13) | 0.4236 | 0.63 (0.42, 0.93) | 0.0079 |
| Daily | 0.80 (0.74, 0.88) | | 1.09 (0.98, 1.21) | | 1.17 (0.85, 1.59) | | 0.81 (0.68, 0.97) | | 1.01 (0.85, 1.20) | | 0.78 (0.63, 0.97) | |
| Non-communicable diseases (Ref: 0 diseases) | | | | | | | | | | | | |
| 1 disease | 1.38 (1.27, 1.51) | <0.0001 | 1.34 (1.19, 1.51) | <0.0001 | 1.07 (0.83, 1.38) | 0.0715 | 1.22 (1.00, 1.48) | <0.0001 | 1.15 (0.97, 1.35) | 0.2434 | 1.29 (1.11, 1.50) | <0.0001 |
| 2 diseases | 1.88 (1.68, 2.10) | | 1.31 (1.11, 1.53) | | 1.21 (0.89, 1.63) | | 1.63 (1.34, 1.99) | | 1.15 (0.93, 1.42) | | 1.85 (1.45, 2.37) | |
| 3+ diseases | 2.17 (1.88, 2.50) | | 1.38 (1.12, 1.70) | | 1.62 (1.11, 2.34) | | 2.62 (2.16, 3.17) | | 0.96 (0.73, 1.24) | | 3.16 (2.01, 5.11) | |

**Table S2.** *Cont.*

| Factors | China | | India | | Mexico | | Russian Federation | | South Africa | | Ghana | |
|---|---|---|---|---|---|---|---|---|---|---|---|---|
| | **OR (95% CI) [a]** | **p [a]** | **OR (95% CI) [a]** | **p [a]** | **OR (95% CI) [a]** | **p [a]** | **OR (95% CI) [a]** | **p [a]** | **OR (95% CI) [a]** | **p [a]** | **OR (95% CI) [a]** | **p [a]** |
| **Health and Health-Risk Factors (*Cont.*)** | | | | | | | | | | | | |
| Pain (Ref: None) | | | | | | | | | | | | |
| Mild | 1.08 (0.99, 1.17) | <0.0001 | 1.03 (0.90, 1.18) | <0.0001 | 1.02 (0.78, 1.34) | <0.0001 | 1.51 (1.28, 1.79) | <0.0001 | 1.32 (1.11, 1.58) | <0.0001 | 1.29 (1.08, 1.55) | <0.0001 |
| Moderate | 1.28 (1.13, 1.43) | | 1.25 (1.08, 1.45) | | 1.50 (1.14, 1.98) | | 2.09 (1.74, 2.51) | | 1.51 (1.26, 1.82) | | 1.63 (1.35, 1.97) | |
| Severe/extreme | 1.64 (1.31, 2.07) | | 1.63 (1.39, 1.91) | | 2.23 (1.56, 3.16) | | 2.44 (1.94, 3.10) | | 1.71 (1.34, 2.17) | | 2.20 (1.77, 2.74) | |
| Self-rated health (Ref: Good/very good) | | | | | | | | | | | | |
| Moderate | 1.14 (1.04, 1.24) | <0.0001 | 0.95 (0.84, 1.08) | <0.0001 | 1.09 (0.86, 1.38) | 0.0009 | 1.44 (1.18, 1.78) | <0.0001 | 1.31 (1.12, 1.54) | <0.0001 | 2.01 (1.74, 2.32) | <0.0001 |
| Bad/very bad | 1.63 (1.47, 1.81) | | 2.12 (1.82, 2.46) | | 1.80 (1.30, 2.47) | | 4.52 (3.55, 5.76) | | 2.28 (1.85, 2.80) | | 3.99 (3.29, 4.86) | |
| **Functional Factors** | | | | | | | | | | | | |
| Mobility (Ref: Mobility) | | | | | | | | | | | | |
| Dismobility | 2.11 (1.78, 2.49) | <0.0001 | 2.05 (1.76, 2.37) | <0.0001 | 2.41 (1.93, 3.02) | <0.0001 | [c] | | [c] | | 1.88 (1.65, 2.15) | <0.0001 |
| Distance vision impairment (Ref: Mild or none) | | | | | | | | | | | | |
| Moderate or greater | 1.35 (1.20, 1.52) | <0.0001 | 1.22 (1.06, 1.40) | 0.0057 | [c] | | [c] | | 1.66 (1.34, 2.05) | <0.0001 | 1.70 (1.40, 2.06) | <0.0001 |
| Near vision impairment (Ref: Mild or none) | | | | | | | | | | | | |
| Moderate or greater | 1.26 (1.17, 1.36) | <0.0001 | 1.01 (0.91, 1.12) | 0.8860 | [c] | | [c] | | 1.08 (0.93, 1.25) | 0.3309 | 1.51 (1.31, 1.74) | <0.0001 |
| Verbal learning and memory | | | | | | | | | | | | |
| Words recalled immediately [d] | 1.04 (1.01, 1.06) | 0.0025 | 0.89 (0.85, 0.92) | <0.0001 | 0.86 (0.80, 0.92) | <0.0001 | 0.84 (0.81, 0.88) | <0.0001 | 0.80 (0.76, 0.84) | <0.0001 | 0.84 (0.80, 0.88) | <0.0001 |
| Words lost with delay [d] | 1.03 (1.00, 1.05) | 0.0225 | 0.99 (0.95, 1.02) | 0.4637 | 0.93 (0.88, 0.98) | 0.0061 | 1.04 (0.99, 1.09) | 0.0869 | [c] | | 1.03 (0.99, 1.07) | 0.1850 |
| IADL [d] | 1.72 (1.58, 1.87) | <0.0001 | 1.45 (1.36, 1.54) | <0.0001 | 1.82 (1.63, 2.03) | <0.0001 | 2.58 (2.30, 2.91) | <0.0001 | 1.48 (1.36, 1.61) | <0.0001 | 1.64 (1.51, 1.79) | <0.0001 |
| ADL [d] | 1.94 (1.75, 2.15) | <0.0001 | 1.56 (1.45, 1.69) | <0.0001 | 2.01 (1.76, 2.30) | <0.0001 | 2.91 (2.55, 3.32) | <0.0001 | 1.60 (1.45, 1.76) | <0.0001 | 1.71 (1.55, 1.89) | <0.0001 |
| Disability [d] | 1.02 (1.02, 1.03) | <0.0001 | 1.02 (1.02, 1.02) | <0.0001 | 1.03 (1.02, 1.04) | <0.0001 | 1.05 (1.04, 1.05) | <0.0001 | 1.02 (1.02, 1.02) | <0.0001 | 1.02 (1.02, 1.03) | <0.0001 |
| Quality of Life [d] | 1.00 (0.99, 1.00) | 0.0115 | 0.98 (0.97, 0.98) | <0.0001 | 0.98 (0.97, 0.99) | <0.0001 | 0.97 (0.97, 0.98) | <0.0001 | 0.97 (0.97, 0.98) | <0.0001 | 0.98 (0.97, 0.98) | <0.0001 |
| **Interpersonal Factors** | | | | | | | | | | | | |
| Emotional loneliness (Ref: No) | | | | | | | | | | | | |
| Yes | 1.09 (0.93, 1.28) | 0.2689 | 1.12 (0.98, 1.29) | 0.1065 | 1.53 (1.17, 1.98) | 0.0014 | 2.24 (1.80, 2.80) | <0.0001 | 1.45 (1.15, 1.82) | 0.0016 | 3.17 (2.54, 3.98) | <0.0001 |
| Friends visiting home (Ref: Never) | | | | | | | | | | | | |
| Once or twice per year | 0.98 (0.90, 1.07) | 0.0690 | 0.86 (0.74, 1.01) | 0.0003 | 0.95 (0.71, 1.27) | 0.3015 | 0.64 (0.51, 0.80) | <0.0001 | 1.25 (0.95, 1.65) | <0.0001 | 0.85 (0.64, 1.12) | 0.0500 |
| Once or twice per month | 0.91 (0.81, 1.01) | | 0.72 (0.62, 0.84) | | 1.13 (0.81, 1.57) | | 0.56 (0.44, 0.70) | | 0.74 (0.59, 0.94) | | 1.05 (0.83, 1.32) | |
| Once per week or more | 1.10 (0.95, 1.28) | | 0.80 (0.69, 0.93) | | 1.26 (0.95, 1.65) | | 0.53 (0.40, 0.71) | | 1.00 (0.81, 1.26) | | 0.85 (0.70, 1.04) | |
| Visiting people (Ref: Never) [e] | | | | | | | | | | | | |
| Once or twice per year | 0.79 (0.72, 0.85) | <0.0001 | 0.68 (0.58, 0.79) | <0.0001 | 0.81 (0.61, 1.07) | 0.1796 | 0.68 (0.58, 0.80) | <0.0001 | 0.82 (0.65, 1.04) | 0.188 | 1.16 (0.93, 1.46) | <0.0001 |
| Once or twice per month | 0.81 (0.72, 0.91) | | 0.54 (0.46, 0.63) | | 0.98 (0.70, 1.35) | | 0.61 (0.50, 0.74) | | 0.80 (0.65, 0.98) | | 0.90 (0.74, 1.10) | |
| Once per week or more | 0.92 (0.78, 1.07) | | 0.50 (0.43, 0.59) | | 1.23 (0.89, 1.68) | | 0.47 (0.33, 0.66) | | 0.85 (0.69, 1.04) | | 0.58 (0.48, 0.70) | |
| Socializing with coworkers (Ref: Never) | | | | | | | | | | | | |
| Once or twice per year | 0.87 (0.78, 0.96) | 0.0002 | 1.11 (0.97, 1.26) | 0.1275 | 0.60 (0.42, 0.85) | 0.0399 | 0.58 (0.49, 0.69) | <0.0001 | [c] | | 0.68 (0.54, 0.85) | <0.0001 |
| Once or twice per month | 0.81 (0.73, 0.90) | | 0.92 (0.79, 1.07) | | 0.90 (0.58, 1.36) | | 0.50 (0.41, 0.61) | | | | 0.40 (0.32, 0.48) | |
| Once per week or more | 0.83 (0.75, 0.92) | | 0.95 (0.82, 1.10) | | 1.05 (0.67, 1.60) | | 0.48 (0.39, 0.60) | | | | 0.54 (0.47, 0.63) | |

**Table S2.** *Cont.*

| Factors | China | | India | | Mexico | | Russian Federation | | South Africa | | Ghana | |
|---|---|---|---|---|---|---|---|---|---|---|---|---|
| | **OR (95% CI)** [a] | **p** [a] | **OR (95% CI)** [a] | **p** [a] | **OR (95% CI)** [a] | **p** [a] | **OR (95% CI)** [a] | **p** [a] | **OR (95% CI)** [a] | **p** [a] | **OR (95% CI)** [a] | **p** [a] |
| **Interpersonal Factors (Cont.)** | | | | | | | | | | | | |
| Social activities outside of home (Ref: Never) | | | | | | | | | | | | |
| Once or twice per year | 0.81 (0.73, 0.89) | <0.0001 | 0.95 (0.83, 1.08) | 0.0003 | 1.31 (1.03, 1.66) | 0.0534 | 0.99 (0.83, 1.17) | 0.0375 | 1.02 (0.83, 1.26) | 0.0421 | 0.92 (0.73, 1.16) | <0.0001 |
| Once or twice per month | 0.94 (0.84, 1.07) | | 0.74 (0.63, 0.87) | | 0.95 (0.68, 1.32) | | 0.80 (0.66, 0.96) | | 1.09 (0.90, 1.32) | | 1.54 (1.24, 1.91) | |
| Once per week or more | 1.42 (1.16, 1.75) | | 0.72 (0.55, 0.93) | | 0.82 (0.53, 1.25) | | 0.82 (0.67, 1.02) | | 1.30 (1.06, 1.60) | | 0.71 (0.58, 0.86) | |
| Marital status (Ref: Coupled) | | | | | | | | | | | | |
| Uncoupled | 1.38 (1.25, 1.53) | <0.0001 | 1.41 (1.25, 1.58) | <0.0001 | 1.27 (1.02, 1.57) | 0.0297 | 1.61 (1.40, 1.85) | <0.0001 | 1.35 (1.18, 1.56) | <0.0001 | 1.42 (1.25, 1.61) | <0.0001 |
| **Environmental Factors** | | | | | | | | | | | | |
| Location (Ref: Urban) | | | | | | | | | | | | |
| Rural | 0.51 (0.48, 0.55) | <0.0001 | 0.93 (0.83, 1.05) | 0.2566 | 0.56 (0.43, 0.72) | <0.0001 | 0.85 (0.73, 0.99) | 0.0392 | 1.05 (0.90, 1.22) | 0.5267 | 0.78 (0.69, 0.89) | 0.0001 |
| Personal motorized transport (Ref: No) | | | | | | | | | | | | |
| Yes | 1.20 (1.11, 1.29) | <0.0001 | 0.82 (0.73, 0.93) | 0.0014 | 0.96 (0.77, 1.20) | 0.7237 | 0.63 (0.55, 0.73) | <0.0001 | 0.63 (0.54, 0.75) | <0.0001 | 0.93 (0.70, 1.22) | 0.5816 |
| Computer in household (Ref: No) | | | | | | | | | | | | |
| Yes | 1.56 (1.43, 1.70) | <0.0001 | 1.00 (0.78, 1.26) | 0.9731 | 1.27 (0.95, 1.68) | 0.0973 | 0.76 (0.64, 0.90) | 0.0013 | 0.70 (0.56, 0.86) | 0.0008 | 0.99 (0.73, 1.34) | 0.9533 |
| Safety out on the street after dark (Ref: Completely/very safe) | | | | | | | | | | | | |
| Moderately safe | 1.07 (0.98, 1.16) | 0.0655 | 0.64 (0.56, 0.72) | <0.0001 | 0.94 (0.70, 1.24) | 0.8513 | 1.04 (0.85, 1.29) | 0.0053 | 0.83 (0.66, 1.05) | <0.0001 | 1.74 (1.47, 2.05) | <0.0001 |
| Slightly safe/not at all | 1.13 (1.01, 1.26) | | 0.58 (0.50, 0.68) | | 1.01 (0.79, 1.29) | | 1.29 (1.06, 1.56) | | 0.60 (0.49, 0.72) | | 1.55 (1.23, 1.95) | |
| Safety when home alone (Ref: Completely/very safe) | | | | | | | | | | | | |
| Moderately safe | 1.06 (0.95, 1.18) | 0.6246 | 0.66 (0.58, 0.75) | <0.0001 | 0.90 (0.68, 1.17) | 0.1866 | 1.07 (0.92, 1.26) | <0.0001 | 0.77 (0.64, 0.94) | <0.0001 | 1.10 (0.90, 1.35) | 0.3430 |
| Slightly safe/not at all | 1.00 (0.80, 1.24) | | 0.70 (0.58, 0.84) | | 0.78 (0.60, 1.02) | | 1.46 (1.22, 1.74) | | 0.68 (0.57, 0.81) | | 1.20 (0.88, 1.64) | |

[a] Odds ratio (95% confidence interval) and p value from a univariate model including each factor separately. [b] Education levels for some countries collapsed (e.g., secondary/high and college or more for India) due to low numbers. [c] Factor omitted from the analyses due to large amounts of missing or implausible data. [d] Higher scores = higher function for words recalled immediately and quality of life; higher scores = lower function for words lost with delay, IADL, ADL, and disability. [e] Visiting people in different neighborhoods or them coming to visit.
